# Racial and Ethnic Survival Disparities Among Children With High-Risk Neuroblastoma: A Children’s Oncology Group Report

**DOI:** 10.1001/jamanetworkopen.2024.58531

**Published:** 2025-02-14

**Authors:** Puja J. Umaretiya, Arlene Naranjo, Fan F. Zhang, Julie R. Park, Brian D. Weiss, Meaghan Granger, Ami V. Desai, M. Fevzi Ozkaynak, Alice L. Yu, Rahela Aziz-Bose, Sandi L. Pruitt, Steven G. DuBois, Rochelle Bagatell, Kira Bona

**Affiliations:** 1 Division of Pediatric Hematology/Oncology, Department of Pediatrics, University of Texas Southwestern Medical Center, Dallas; 2 Harold C. Simons Comprehensive Cancer Center, University of Texas Southwestern Medical Center, Dallas; 3 Children’s Oncology Group Statistics and Data Center, Department of Biostatistics, University of Florida, Gainesville; 4 Children’s Oncology Group Statistics and Data Center, Monrovia, California; 5 Department of Oncology, St Jude Children’s Research Hospital, Memphis, Tennessee; 6 Division of Hematology/Oncology/Stem Cell Transplant, Department of Pediatrics, Indiana University School of Medicine, Riley Children’s Health, Indianapolis; 7 Department of Pediatrics, Section of Hematology/Oncology/Stem Cell Transplantation, University of Chicago Medical Center, Chicago, Illinois; 8 Maria Fareri Children’s Hospital Westchester Medical Center, New York Medical College, Valhalla; 9 Department of Pediatrics, University of California, San Diego; 10 Chang Gung Memorial Hospital, Taoyuan, Taiwan; 11 Department of Pediatric Oncology, Dana-Farber Cancer Institute, Boston, Massachusetts; 12 Boston Children’s Hospital, Division of Pediatric Hematology/Oncology, Boston, Massachusetts; 13 Harvard Medical School, Boston, Massachusetts; 14 Division of Population Sciences, Dana-Farber Cancer Institute, Boston, Massachusetts; 15 Peter O’Donnell Jr. School of Public Health, University of Texas Southwestern Medical Center, Dallas; 16 Department of Pediatrics, Children’s Hospital of Philadelphia and Perelman School of Medicine, University of Pennsylvania, Philadelphia

## Abstract

**Importance:**

Whether population-based racial and ethnic survival disparities for children with high-risk neuroblastoma persist in the clinical trial setting is unknown.

**Objective:**

To investigate racial and ethnic survival disparities among children with high-risk neuroblastoma treated on frontline clinical trials.

**Design, Setting, and Participants:**

This retrospective cohort study used data from Children’s Oncology Group (COG) high-risk neuroblastoma trials from January 1, 2007, to December 31, 2016, with a data freeze on June 30, 2021. Children with high-risk neuroblastoma were analyzed in 2 cohorts: induction/consolidation trial participants and post-consolidation trial participants. Statistical analyses were performed from September 2, 2021, to December 30, 2024.

**Exposures:**

Race and ethnicity were the primary exposures, categorized as Hispanic, non-Hispanic Black, non-Hispanic other (American Indian or Alaska Native, Asian, and Native Hawaiian or Other Pacific Islander), or non-Hispanic White.

**Main Outcomes and Measures:**

Primary outcomes included overall survival (OS) and event-free survival (EFS) from time of trial enrollment, estimated by Kaplan-Meier methods. Associations with race and ethnicity were evaluated by log-rank tests and Cox proportional hazards regression models. Secondary outcomes included induction delays, early trial withdrawal, relapse as first event, death as first event, postrelapse OS, and early phase trial enrollment.

**Results:**

The induction/consolidation cohort (median follow-up, 8.3 years [IQR, 6.1-9.8 years]) included 696 patients (404 males [58.1%]; 79 Hispanic patients [11.4%], 109 non-Hispanic Black patients [15.7%], 27 patients of non-Hispanic other race [3.9%], and 481 non-Hispanic White patients [69.1%]). The post-consolidation cohort (median follow-up, 7.5 years [IQR, 5.8-9.4 years]) included 935 patients (567 males [60.6%]; 87 Hispanic patients [9.3%], 145 non-Hispanic Black patients [15.5%], 41 patients of non-Hispanic other race [4.4%], and 662 non-Hispanic White patients [70.8%]). In multivariable Cox proportional hazards regression models, Hispanic children experienced significantly inferior OS (hazard ratio [HR], 1.78; 95% CI, 1.25-2.53; *P* = .01) on induction/consolidation studies compared with non-Hispanic White children; EFS did not differ. Non-Hispanic Black (HR, 1.54; 95% CI, 1.13-2.11) and Hispanic children (HR, 1.63; 95% CI, 1.09-2.43) experienced inferior OS on post-consolidation studies compared with non-Hispanic White children (*P* = .009); Hispanic children in post-consolidation studies experienced inferior EFS (HR, 1.68; 95% CI, 1.14-2.47; *P* = .02). Death as first event and postrelapse OS also differed by race and ethnicity.

**Conclusions and Relevance:**

This study suggests that Black and Hispanic children with high-risk neuroblastoma experienced inferior OS despite uniform planned treatment on frontline COG clinical trials. Investigated mechanisms did not completely explain survival disparities. Future evaluation of disparate treatment-related toxicities and postrelapse care as explanatory mechanisms are key next steps to promote equity.

## Key Points

**Question:**

Are there survival disparities by race and ethnicity among children with high-risk neuroblastoma treated on frontline Children’s Oncology Group (COG) trials?

**Findings:**

Among a cohort of 696 patients on induction/consolidation studies, Hispanic children had a 78% higher hazard of death compared with non-Hispanic White children. Among a cohort of 935 patients on post-consolidation studies, Black children had a 54% higher hazard of death, and Hispanic children had a 63% higher hazard of death compared with non-Hispanic White children.

**Meaning:**

This study suggests that Black and Hispanic children with high-risk neuroblastoma experienced inferior overall survival despite receiving highly standardized treatment on frontline COG clinical trials.

## Teaser

This cohort study uses data from frontline Children’s Oncology Group trials to investigate racial and ethnic survival disparities among children with high-risk neuroblastoma.

## Introduction

Children who are Black, Hispanic, or living in poverty experience inferior survival across many childhood cancers.^1,2,3,4,5,6,7,8,9,10,11,12,13,14^ Population-based data have been instrumental in identifying these disparities, but have lacked robust clinical data necessary to identify factors associated with the observed disparities. A substantial proportion of children with cancer receive initial treatment on clinical trials.^15^ Therefore, leveraging trial data to evaluate potential mechanisms underlying racial and ethnic survival disparities is a key next step to improving survival for marginalized children with cancer.

Neuroblastoma, the most common extracranial pediatric solid tumor, lends itself to the study of mechanisms underlying survival disparities given evidence of trial-based disparities in this disease and high reliance on a clinical trial model of care. Prior Children’s Oncology Group (COG) studies demonstrated a higher likelihood of late relapse among Black children compared with White children enrolled on the biology study ANBL00B1^3^ and survival disparities among children living in poverty receiving post-consolidation immunotherapy.^6^ Mechanisms for disparate outcomes despite uniform planned trial treatment remain unknown, limiting opportunities for intervention.

We aimed to examine associations of race and ethnicity with survival among children with high-risk neuroblastoma treated on frontline COG trials. We leveraged this trial-based cohort to explore potential care delivery mechanisms that may be associated with disparate outcomes.

## Methods

### Study Design and Cohort

**Figure 1.**
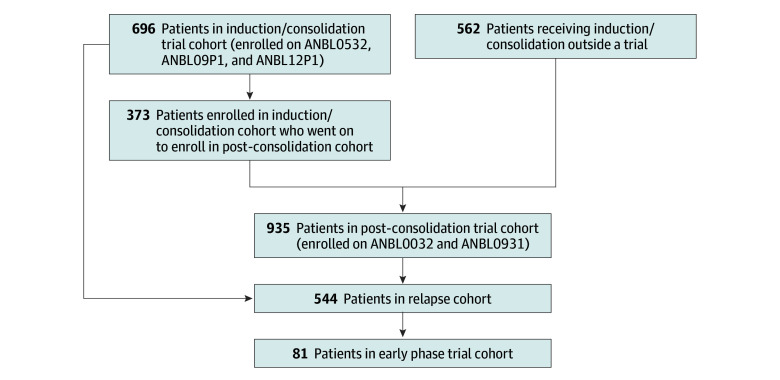
Patient Flow Diagram for Analytic Cohorts

This retrospective cohort study included patients younger than 30 years with high-risk neuroblastoma enrolled on ANBL00B1 and frontline COG phase 3 or pilot trials from January 1, 2007, to December 31, 2016, with a data freeze on June 30, 2021. Patients at non-US centers and those missing race or ethnicity or insurance data were excluded (n = 810). Disease-associated characteristics were not different between included and excluded patients (eTable 1 in Supplement 1). The analytic cohort included patients enrolled on (1) induction/consolidation trials ANBL0532, ANBL09P1, and ANBL12P1 and (2) post-consolidation immunotherapy trials ANBL0032 and ANBL0931 (Figure 1). The Pediatric Central institutional review board or participating institution institutional review boards approved original trial protocols. Written informed consent was obtained from participants and/or guardians at trial enrollment. This study followed the Strengthening the Reporting of Observational Studies in Epidemiology (STROBE) reporting guideline.

Primary outcomes of individual trials were previously published (eTable 2 in Supplement 1).^16,17,18,19,20^ Children enrolled on frontline induction/consolidation and post-consolidation trials were analyzed separately. Patients with relapsed or progressive disease in either cohort were combined to create a relapse cohort for additional analyses (Figure 1), including evaluation of subsequent enrollment on COG early phase trials. Early phase trials included all COG phase 1, 2, or 1/2 trials open in 2006-2017 for patients with relapsed high-risk neuroblastoma (eTable 3 in Supplement 1).

### Exposure Variable

Race and ethnicity were the primary exposures of interest, obtained from trial case report forms collected per US Office of Management and Budget guidelines. Data were categorized as Hispanic, non-Hispanic Black (hereafter, *Black*), non-Hispanic other (American Indian or Alaska Native, Asian, and Native Hawaiian or Other Pacific Islander; hereafter, *other*), and non-Hispanic White (hereafter, *White*). All patients of Hispanic ethnicity were assigned to the Hispanic group. All multiracial patients who listed Black race but not Hispanic ethnicity were assigned to the Black group. All other multiracial patients were assigned to the Other group for statistical modeling due to small sample size. Race and ethnicity categories were combined and a priori categorized to prioritize groups most affected by structural racism in the US.^21^

### Covariates

Trial-collected sociodemographic variables and disease-associated characteristics included age at diagnosis, sex, tumor *MYCN* status, tumor histology, International Neuroblastoma Staging System (INSS) stage, and end-of-induction (EOI) disease response. Socioeconomic status (SES) was proxied at the rural, household, and area levels. Household poverty exposure was proxied by public (Medicaid or Children’s Health Insurance Program [CHIP]) vs private or other (commercial or dual commercial and public, military, and other) insurance at time of first trial enrollment; patients with only public insurance were characterized as household poverty exposed.^6^ Area-level poverty was proxied based on zip codes; patients living in a zip code with 20% or more of the population below the federal poverty level were characterized as area poverty exposed.^22^ Rural and urban status was similarly proxied by zip codes that were dichotomized as urban or rural based on 2010 US Census linked to United States Department of Agriculture Rural Urban Commuting Area codes.^23,24^

### Primary Outcomes

Event-free survival (EFS) and OS were the primary outcomes of interest. EFS was measured as time from study enrollment until first occurrence of relapse, progressive disease, secondary malignant neoplasm, or death, or censored at last contact if no event occurred. OS was measured as time from study enrollment until death or censored at last contact.

### Secondary Outcomes

Secondary outcomes focused on a priori care delivery inflection points hypothesized to mechanistically underlie disparate survival, including (1) induction delay, (2) early trial withdrawal, (3) relapse or progression as a first event, and (4) early phase trial enrollment among patients experiencing relapse or progression. We additionally examined death as a first event and OS after first relapse. Induction delay was proxied using median length of induction only for patients enrolled on induction/consolidation trial ANBL0532. The proportion of patients discontinuing trial participation during induction for reasons other than progression was assessed separately for patients on ANBL0532 (groupwide trial) and ANBL09P1 (limited institution trial)/ANBL12P1 (groupwide trial).

We examined the proportion of patients experiencing relapse or progression as a first event after enrollment in both cohorts. For patients who experienced relapse or progression, subsequent enrollment on COG early phase trials was evaluated based on race and ethnicity and proxied SES.

### Statistical Analysis

Statistical analyses were performed from September 2, 2021, to December 30, 2024. Demographic and clinical features were summarized descriptively by race and ethnicity and compared using χ^2^ tests^25^ or Fisher exact tests^26^ for categorical variables and Kruskal-Wallis tests^27^ for continuous variables. OS and EFS were estimated using Kaplan-Meier methods with SEs.^28,29^ Associations between race and ethnicity and survival outcomes were evaluated with log-rank tests.^30^ Multivariable Cox proportional hazards regression models of survival outcomes included all statistically significant univariate Cox proportional hazards regression model factors (*P* < .05) and used a backward selection process.^31,32^ Cumulative incidence of relapse or death as first event was compared with the Gray test.^33^

Frequency of relapse and care delivery inflection points, including induction delays and early trial withdrawal for nonprogression, were summarized descriptively and compared by race and ethnicity using χ^2^ tests or Fisher exact tests for categorical variables and Kruskal-Wallis tests for continuous variables. Associations between race and ethnicity and early phase trial enrollment for patients who relapsed were evaluated with a χ^2^ test. Multivariable logistic regression models^34^ compared early phase trial enrollment by race and ethnicity adjusted for significant univariate factors (*P* < .05) using a Wald test.^35^

Exploratory analyses considering proxied SES as the primary exposure of interest were conducted for each outcome (eMethods/eResults, eTables 4-7, and eFigures 1-3 in Supplement 1) and significant findings are included below. Statistical testing was performed with SAS, version 9.4 (SAS Institute Inc). All *P* values were from 2-sided tests and results were deemed statistically significant at *P* < .05.

## Results

### Frontline Trial Patient Characteristics

#### Induction/Consolidation Cohort (ANBL0532, ANBL09P1, and ANBL12P1)

The induction/consolidation cohort included 696 patients (404 males [58.1%] and 292 females [42.0%]; 109 Black patients [15.7%], 79 Hispanic patients [11.4%], 27 patients of other race [3.9%], and 481 White patients [69.1%]) (Table 1). There were no differences in age, sex, *MYCN* amplification, tumor histology, INSS stage, or EOI response by race and ethnicity. Hispanic (58.2% [46 of 79]) and Black (50.5% [55 of 109]) patients were disproportionately household poverty exposed compared with White patients (25.2% [121 of 481]; *P* < .001). Black (51.4% [56 of 109]) and Hispanic patients (41.8% [33 of 79]) were disproportionately area-level poverty exposed compared with White patients (18.4% [88 of 481]; *P* < .001). White patients were more likely to live in rural areas (18.3% [88 of 481]; *P* = .004).

**Table 1.** Characteristics of Trial Patients by Race and Ethnicity

Characteristic	Overall	White	Black	Hispanic	Other^a^	*P* value
**Induction/consolidation studies (ANBL0532, ANBL09P1, and ANBL12P1)**						
No. (%)	696	481 (69.1)	109 (15.7)	79 (11.4)	27 (3.9)	NA
Sociodemographic characteristics						
Age, No. (%)						
<18 mo	86 (12.4)	68 (14.1)	10 (9.2)	5 (6.3)	3 (11.1)	.16
≥18 mo	610 (87.6)	413 (85.9)	99 (90.8)	74 (93.7)	24 (88.9)	
Sex, No. (%)						
Male	404 (58.1)	276 (57.4)	70 (64.2)	42 (53.2)	16 (59.3)	.46
Female	292 (42.0)	205 (42.6)	39 (35.8)	37 (46.8)	11 (40.7)	
Household poverty, No. (%)						
No	467 (67.1)	360 (74.8)	54 (49.5)	33 (41.8)	20 (74.1)	<.001
Yes (public insurance)	229 (32.9)	121 (25.2)	55 (50.5)	46 (58.2)	7 (25.9)	
Area poverty, No. (%)^b^						
No	513 (74.0)	390 (81.6)	53 (48.6)	46 (58.2)	24 (88.9)	<.001
Yes (≥20% of zip code population living below FPL)	180 (26.0)	88 (18.4)	56 (51.4)	33 (41.8)	3 (11.1)	
Geographic location, No. (%)^b^						
Rural	106 (15.3)	88 (18.3)	12 (11.0)	3 (3.8)	3 (11.1)	.004
Urban	589 (84.7)	392 (81.7)	97 (89.0)	76 (96.2)	24 (88.9)	
Disease characteristics						
Tumor *MYCN* status, No. (%)^b^						
Amplified	272 (43.3)	193 (44.5)	33 (33.7)	33 (47.1)	13 (50.0)	.18
Not amplified	356 (56.7)	241 (55.5)	65 (66.3)	37 (52.9)	13 (50.0)	
Tumor histology, No. (%)^b^						
Unfavorable	603 (96.3)	420 (96.1)	89 (95.7)	71 (97.3)	23 (100.0)	.93^c^
Favorable	23 (3.7)	17 (3.9)	4 (4.3)	2 (2.7)	0	
INSS stage, No. (%)						
1	1 (0.1)	1 (0.2)	0	0	0	.69^c^^,^^d^
2A	2 (0.3)	2 (0.4)	0	0	0	
2B	6 (0.9)	4 (0.8)	2 (1.8)	0	0	
3	71 (10.2)	49 (10.2)	7 (6.4)	11 (13.9)	4 (14.8)	
4	614 (88.2)	424 (88.2)	99 (90.8)	68 (86.1)	23 (85.2)	
4S	2 (0.3)	1 (0.2)	1 (0.9)	0	0	
EOI disease response, No. (%)^b^^,^^e^						
CR	113 (18.5)	86 (20.4)	11 (11.6)	11 (15.1)	5 (21.7)	.15^e^
VGPR	148 (24.2)	103 (24.5)	19 (20.0)	20 (27.4)	6 (26.1)	
PR	207 (33.8)	141 (33.5)	40 (42.1)	18 (24.7)	8 (34.8)	
MR	56 (9.2)	34 (8.1)	11 (11.6)	10 (13.7)	1 (4.3)	
NR	33 (5.4)	24 (5.7)	4 (4.2)	3 (4.1)	2 (8.7)	
PD	55 (9.0)	33 (7.8)	10 (10.5)	11 (15.1)	1 (4.3)	
Trial, No. (%)						
ANBL0532	496 (71.3)	345 (71.7)	75 (68.8)	62 (78.5)	14 (51.9)	.10
ANBL09P1 (limited)	82 (11.8)	58 (12.1)	16 (14.7)	4 (5.1)	4 (14.8)	
ANBL12P1	118 (17.0)	78 (16.2)	18 (16.5)	13 (16.5)	9 (33.3)	
**Post-consolidation studies (ANBL0032 and ANBL0931)**						
No. (%)	935	662 (70.8)	145 (15.5)	87 (9.3)	41 (4.4)^f^	
Sociodemographic characteristics						
Age, No. (%)						
<18 mo	145 (15.5)	106 (16.0)	22 (15.2)	11 (12.6)	6 (14.6)	.87
≥18 mo	790 (84.5)	556 (84.0)	123 (84.8)	76 (87.4)	35 (85.4)	
Sex, No. (%)						
Male	567 (60.6)	403 (60.9)	91 (62.8)	50 (57.5)	23 (56.1)	.80
Female	368 (39.4)	259 (39.1)	54 (37.2)	37 (42.5)	18 (43.9)	
Household poverty (insurance), No. (%)						
No	647 (69.2)	510 (77.0)	74 (51.0)	37 (42.5)	26 (63.4)	<.001
Yes (public insurance)	288 (30.8)	152 (23.0)	71 (49.0)	50 (57.5)	15 (36.6)	
Area poverty, No. (%)^g^						
No	707 (76.0)	548 (83.2)	74 (51.4)	52 (60.5)	33 (80.5)	<.001
Yes (≥20% of zip code population living below FPL)	223 (24.0)	111 (16.8)	70 (48.6)	34 (39.5)	8 (19.5)	
Geographic location, No. (%)^g^						
Rural	143 (15.3)	115 (17.4)	14 (9.7)	8 (9.3)	6 (14.6)	.04
Urban	791 (84.7)	547 (82.6)	131 (90.3)	78 (90.7)	35 (85.4)	
Disease characteristics						
Tumor *MYCN* status, No. (%)^g^						
Amplified	325 (45.7)	236 (46.6)	46 (38.3)	29 (50.0)	14 (51.9)	.30
Not amplified	386 (54.3)	270 (53.4)	74 (61.7)	29 (50.0)	13 (48.1)	
Tumor histology, No. (%)^g^						
Unfavorable	657 (94.1)	470 (94.0)	106 (93.0)	55 (94.8)	26 (100.0)	.70^c^
Favorable	41 (5.9)	30 (6.0)	8 (7.0)	3 (5.2)	0	
INSS stage, No. (%)^g^						
1	4 (0.5)	4 (0.7)	0	0	0	.42^d^
2A	5 (0.6)	5 (0.9)	0	0	0	
2B	19 (2.4)	16 (2.8)	2 (1.5)	1 (1.4)	0	
3	102 (12.8)	71 (12.6)	15 (11.3)	13 (18.8)	3 (10.0)	
4	657 (82.4)	461 (81.6)	114 (85.7)	55 (79.7)	27 (90.0)	
4S	10 (1.3)	8 (1.4)	2 (1.5)	0	0	
EOI disease response, No. (%)^e^^,^^g^						
CR	260 (29.8)	199 (32.5)	31 (22.5)	23 (27.7)	7 (18.0)	.45^c^^,^^e^
VGPR	273 (31.3)	189 (30.8)	41 (29.7)	27 (32.5)	16 (41.0)	
PR	322 (36.9)	214 (34.9)	62 (44.9)	30 (36.1)	16 (41.0)	
MR	13 (1.5)	7 (1.1)	3 (2.2)	3 (3.6)	0	
NR	1 (0.1)	0	1 (0.7)	0	0	
PD	4 (0.5)	4 (0.7)	0	0	0	
Trial, No. (%)						
ANBL0032						.56 (ANBL0032 pre-2009 vs ANBL0032 post-2009 vs ANBL0931)
Pre-2009 (randomization)	62 (6.6)	49 (7.4)	7 (4.8)	4 (4.6)	2 (4.9)	
Immunotherapy	33 (3.5)	26 (3.9)	4 (2.8)	3 (3.4)	0	
Cis-RA	29 (3.1)	23 (3.5)	3 (2.1)	1 (1.1)	2 (4.9)	
Post-2009	793 (84.8)	551 (83.2)	130 (89.7)	76 (87.4)	36 (87.8)	
ANBL0931 (limited)	80 (8.6)	62 (9.4)	8 (5.5)	7 (8.1)	3 (7.3)	

Abbreviations: Cis-RA, 13-cis-retinoic acid; CR, complete response; EOI, end-of-induction; FPL, federal poverty level; INSS, International Neuroblastoma Staging System; MR, mixed response; NA, not applicable; NR, no response; PD, progressive disease; PR, partial response; VGPR, very good partial response.

^a^ Other racial group for the induction/consolidation cohort includes American Indian or Alaska Native (n = 1), Asian (n = 25), and Native Hawaiian or Other Pacific Islander (n = 1).

^b^ Three with unknown area-level poverty; 1 with unknown geographic location; 68 with unknown *MYCN* status; 70 with unknown tumor histology; 84 with unknown EOI response.

^c^ Fisher exact test was used.

^d^ Non–stage 4 vs stage 4.

^e^ Partial response or better vs less than PR (MR, NR, PD).

^f^ Other racial group for the post-consolidation cohort includes American Indian or Alaska Native (n = 3), Asian (n = 36), and Native Hawaiian or Other Pacific Islander (n = 2).

^g^ Five with unknown area-level poverty; 1 with unknown geographic location; 224 with unknown *MYCN* status; 237 with unknown tumor histology; 138 with unknown INSS stage; 62 with unknown EOI response.

#### Post-Consolidation Cohort (ANBL0032 and ANBL0931)

The post-consolidation cohort included 935 patients (567 males [60.6%] and 368 females [39.4%]; 145 Black patients [15.5%], 87 Hispanic patients [9.3%], 41 patients of other race [4.4%], and 662 White patients [70.8%]) (Table 1); 373 patients were concurrently in the induction/consolidation cohort (Figure 1). There were no differences in age, sex, *MYCN* amplification, tumor histology, INSS stage, or EOI response by race and ethnicity. Associations between race and ethnicity and poverty and rurality measures paralelled the induction/consolidation cohort.

### Associations Between Race and Ethnicity and Survival in Frontline Trials

#### Induction/Consolidation Cohort Survival Analyses

**Figure 2.**
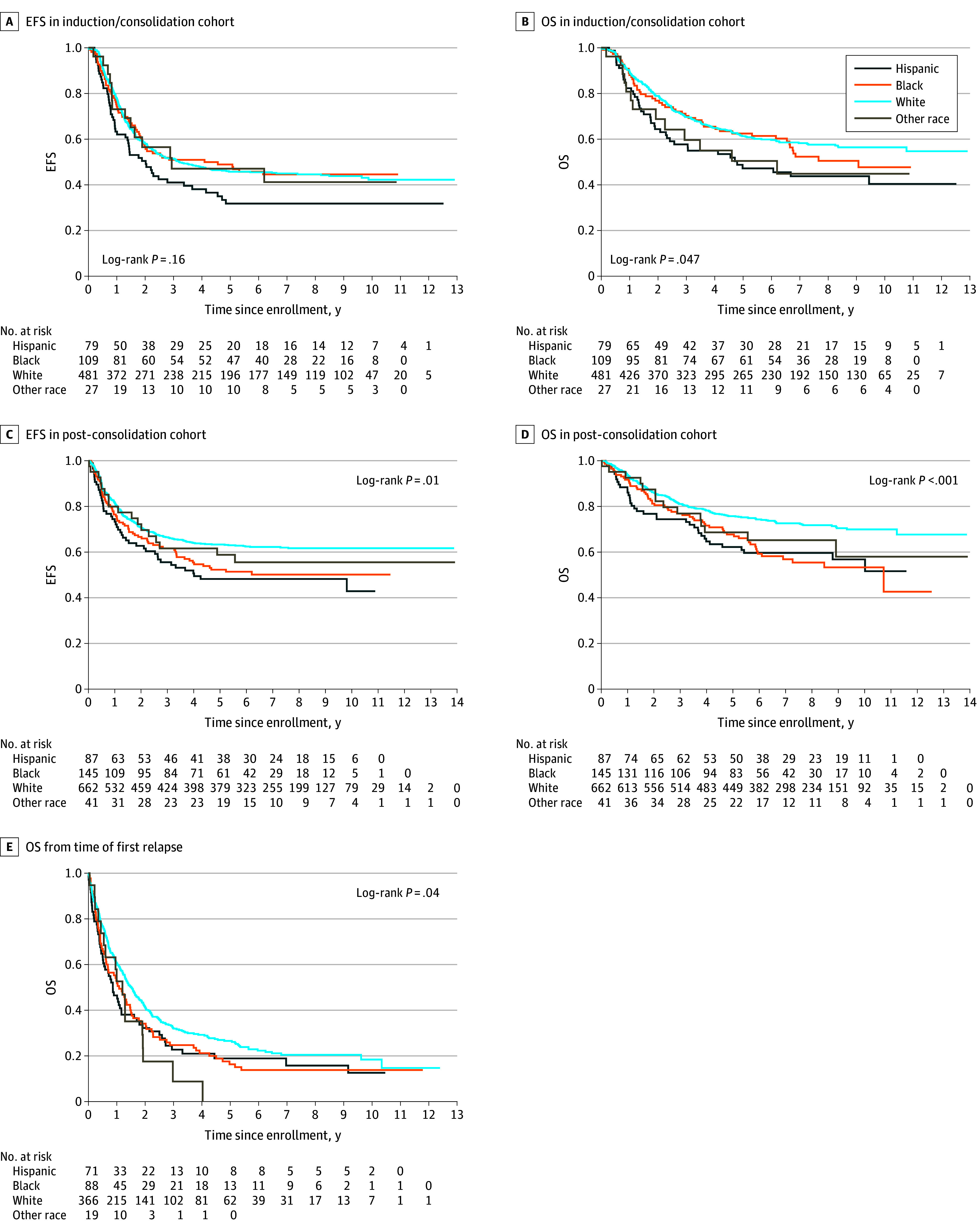
Survival Curves for Induction/Consolidation Cohort, Post-Consolidation Cohort, and Relapse Cohort by Race and Ethnicity A, Event-free survival (EFS) in induction/consolidation cohort from time of enrollment at initial diagnosis. B, Overall survival (OS) in induction/consolidation cohort from time of enrollment at initial diagnosis. C, Post-consolidation EFS from time of enrollment at start of post-consolidation. D, Post-consolidation OS from time of enrollment at start of post-consolidation. E, Overall survival from time of first relapse.

The median follow-up for the 307 patients without an event was 8.3 years (IQR, 6.1-9.8 years). In univariate analyses, EFS did not differ by race and ethnicity (Figure 2A). Five-year OS (SE) for Hispanic patients was inferior to that for White patients (47.2% [6.3%] vs 61.3% [2.3%]; *P* = .047; hazard ratio [HR], 1.55; 95% CI, 1.11-2.15) (Table 2; Figure 2B).

**Table 2.** Survival Data for the Induction/Consolidation and Post-Consolidation Trials

Characteristic	Univariate analyses of outcome				Multivariable analyses of outcome^a^			
	Event-free survival		Overall survival		Event-free survival		Overall survival	
	HR (95% CI)	*P* value	HR (95% CI)	*P* value	HR (95% CI)	*P* value	HR (95% CI)	*P* value
**Induction/consolidation trials (ANBL0532, ANBL09P1, and ANBL12P1)**								
Race and ethnicity								
White	1 [Reference]	.17	1 [Reference]	.049	NA	NA	1 [Reference]	.01
Black	0.99 (0.75-1.32)		1.13 (0.83-1.54)		NA	NA	0.98 (0.69-1.40)	
Hispanic	1.40 (1.04-1.88)		1.55 (1.11-2.15)		NA	NA	1.78 (1.25-2.53)	
Other^b^	1.02 (0.60-1.74)		1.45 (0.83-2.55)		NA	NA	1.45 (0.76-2.74)	
Household poverty (insurance), No. (%)								
No	1 [Reference]	.14	1 [Reference]	.04	NA	NA	1 [Reference]	ELIM^a^
Yes	1.17 (0.95-1.44)		1.28 (1.01-1.62)		NA	NA	ELIM (first; *P* = .67)	
Area poverty, No. (%)								
No	1 [Reference]	.14	1 [Reference]	.05	NA	NA	1 [Reference]	ELIM
Yes (≥20% of zip code population living below FPL)	1.18 (0.95-1.48)		1.28 (1.00-1.64)		NA	NA	ELIM (second; *P* = .25)	
Geographic location, No. (%)								
Rural	1 [Reference]	.76	1 [Reference]	.50	NA	NA	NA	NA
Urban	1.04 (0.79-1.37)		1.12 (0.81-1.54)		NA	NA	NA	NA
Age								
<18 mo	1 [Reference]	.82	1 [Reference]	.16	NA	NA	NA	NA
≥18 mo	0.97 (0.71-1.31)		0.79 (0.57-1.10)					
Sex								
Male	0.97 (0.79-1.18)	.75	1.08 (0.86-1.35)	.53	NA	NA	NA	NA
Female	1 [Reference]		1 [Reference]		NA	NA	NA	NA
Tumor *MYCN* status								
Not amplified	1 [Reference]	.95	1 [Reference]	.01	NA	NA	1 [Reference]	.001
Amplified	1.01 (0.82-1.24)		1.35 (1.06-1.70)		NA	NA	1.52 (1.18-1.95)	
Tumor histology								
Favorable	1 [Reference]	.11	1 [Reference]	.03	NA	NA	1 [Reference]	.02
Unfavorable	1.78 (0.88-3.59)		2.95 (1.10-7.93)		NA	NA	3.23 (1.20-8.68)	
INSS stage								
Non–stage 4	1 [Reference]	<.001	1 [Reference]	<.001	1 [Reference]	<.001	1 [Reference]	<.001
Stage 4	2.45 (1.63-3.67)		2.76 (1.67-4.57)		2.45 (1.63-3.67)		3.21 (1.92-5.37)	
**Post-consolidation trials** (**ANBL0032 and ANBL0931)**								
Race and ethnicity								
White	1 [Reference]	.01	1 [Reference]	<.001	1 [Reference]	.02	1 [Reference]	.009
Black	1.37 (1.05-1.79)		1.65 (1.23-2.22)		1.29 (0.95-1.75)		1.54 (1.13-2.11)	
Hispanic	1.56 (1.14-2.15)		1.73 (1.21-2.47)		1.68 (1.14-2.47)		1.63 (1.09-2.43)	
Other	1.17 (0.72-1.91)		1.39 (0.81-2.39)		0.69 (0.34-1.40)		0.86 (0.42-1.76)	
Household poverty (insurance), No. (%)								
No	1 [Reference]	.10	1 [Reference]	.02	NA	NA	1 [Reference]	ELIM
Yes	1.20 (0.97-1.48)		1.32 (1.04-1.69)		NA	NA	ELIM (first; *P* = .49)	
Neighborhood poverty, No. (%)								
No	1 [Reference]	.53	1 [Reference]	.15	NA	NA	NA	NA
Yes (≥20% of zip code population living below FPL)	1.08 (0.85-1.36)		1.21 (0.93-1.58)		NA	NA	NA	NA
Geographic location, No. (%)								
Rural	1 [Reference]	.80	1 [Reference]	.37	NA	NA	NA	NA
Urban	0.96 (0.73-1.27)		1.16 (0.83-1.63)		NA	NA	NA	NA
Age								
<18 mo	1 [Reference]	.09	1 [Reference]	.24	NA	NA	NA	NA
≥18 mo	1.30 (0.96-1.78)		1.23 (0.87-1.75)		NA	NA	NA	NA
Sex								
Male	1.20 (0.97-1.47)	.10	1.08 (0.85-1.37)	.55	NA	NA	NA	NA
Female	1 [Reference]		1 [Reference]		NA	NA	NA	NA
Tumor *MYCN* status								
Not amplified	1 [Reference]	.91	1 [Reference]	.37	NA	NA	NA	NA
Amplified	0.99 (0.78-1.25)		1.13 (0.87-1.47)		NA	NA	NA	NA
Tumor histology								
Favorable	1 [Reference]	.01	1 [Reference]	.11	1 [Reference]	.008	NA	NA
Unfavorable	2.61 (1.23-5.53)		1.86 (0.88-3.96)		2.75 (1.30-5.83)		NA	NA
INSS stage								
Non–stage 4	1 [Reference]	<.001	1 [Reference]	<.001	1 [Reference]	<.001	1 [Reference]	<.001
Stage 4	2.29 (1.58-3.32)		2.40 (1.55-3.72)		2.29 (1.55-3.38)		2.20 (1.41-3.42)	
EOI disease response								
CR	1 [Reference]	.003	1 [Reference]	.005	1 [Reference]	.04	1 [Reference]	.046
VGPR	1.33 (1.02-1.72)		1.54 (1.14-2.08)		1.39 (1.03-1.89)		1.43 (1.03-1.99)	
PR	1.56 (1.21-2.01)		1.59 (1.18-2.14)		1.44 (1.06-1.97)		1.48 (1.06-2.05)	

Abbreviations: CR, complete response; ELIM, covariate eliminated from backward selection model; EOI, end-of-induction; FPL, federal poverty level; INSS, International Neuroblastoma Staging System; HR, hazard ratio; NA, not applicable; PR, partial response; VGPR, very good partial response.

^a^ Using backward selection starting with all corresponding statistically significant (*P* < .05) univariate factors.

^b^ Other race group includes American Indian or Alaska Native, Asian, and Native Hawaiian or Other Pacific Islander.

In multivariable analyses adjusting for *MYCN* amplification, tumor histology, and INSS stage, Hispanic patients had significantly inferior OS (HR, 1.78, 95% CI, 1.25-2.53; *P* = .01) compared with White patients (Table 2). Household-level poverty was associated with inferior OS in univariate analyses but not the multivariable model; the remainder of proxied SES covariates were not associated with EFS or OS.

#### Post-Consolidation Cohort Survival Analyses

The median follow-up for the 557 patients without an event was 7.5 years (IQR, 5.8-9.4 years). In univariate analyses for EFS, non-Hispanic Black and Hispanic patients experienced significantly inferior EFS (*P* = .01). EFS (SE) at 5 years was 52.3% (4.6%) (HR, 1.37; 95% CI, 1.05-1.79) and 48.2% (5.6%) (HR, 1.56; 95% CI, 1.14-2.15) for Black and Hispanic patients, respectively, compared with 63.3% (2.0%) for Non-Hispanic White patients (Table 2; Figure 2C). In multivariable analyses of EFS adjusting for tumor histology, INSS stage, and EOI disease response, Hispanic patients experienced significantly inferior EFS compared with White patients (HR, 1.68; 95% CI, 1.14-2.47; *P* = .02) (Table 2).

In univariate analyses, Black and Hispanic patients experienced significantly inferior 5-year OS (SE) compared with White patients (Black patients, 67.7% [4.2%]; HR, 1.65; 95% CI, 1.23-2.22; Hispanic patients, 62.2% [5.4%]; HR, 1.73; 95% CI, 1.21-2.47; and White patients, 75.7% [1.8%]; *P* < .001) (Table 2; Figure 2D). In multivariable analyses of OS adjusting for stage and EOI response, Black (HR, 1.54; 95% CI, 1.13-2.11) and Hispanic patients (HR, 1.63; 95% CI, 1.09-2.43) experienced significantly inferior OS (*P* = .009) compared with White patients (Table 2). Household-level poverty was associated with inferior OS in univariate analyses but not in the multivariable model; the remainder of proxied SES covariates were not associated with EFS or OS.

### Potential Care Delivery Mechanisms Underlying Survival Outcome Disparities

Care delivery inflection points were evaluated as potential mechanisms for disparate survival outcomes.

#### Delays During Induction Chemotherapy

The median length of induction for patients in ANBL0532 was 168 days (IQR, 155-179 days). There were no significant differences in induction duration by race and ethnicity (Table 3).

**Table 3.** Potential Mechanisms for Disparate Survival by Race and Ethnicity

Mechanism	Overall	White	Black	Hispanic	Other	*P* value
**Induction/consolidation cohort**						
No.	696	481	109	79	27	NA
Trial withdrawal for reasons other than progression during induction (ANBL0532), No. (%)	184 (37.1)	131 (38.0)	27 (36.0)	20 (32.3)	6 (42.9)	.81
Trial withdrawal for reasons other than progression during induction (ANBL09P1, ANBL12P1), No. (%)	74 (37.0)	49 (36.0)	13 (38.2)	7 (41.2)	5 (38.5)	.98
ANBL0532 total induction length, median (IQR) [No.], days to complete induction	168 (155-179) [479]	167 (155-179) [332]	169 (155-181) [72]	169 (151-181) [61]	166 (159-180) [14]	.91^a^
5-y Cumulative incidence of relapse (as first event), % (SE)	48.5 (1.9)	48.1 (2.3)	46.5 (4.9)	58.7 (5.8)	32.4 (9.8)	.13^b^
5-y Cumulative incidence of death (as first event), % (SE)	6.5 (0.9)	5.9 (1.1)	4.6 (2.0)	9.5 (3.5)	16.2 (7.7)	.049^b^
**Post-consolidation cohort**						
No.	935	662	145	87	41	NA
5-y Cumulative incidence of relapse (as first event), % (SE)	37.5 (1.6)	35.5 (1.9)	41.1 (4.2)	48.2 (5.5)	36.2 (7.9)	.14^b^
5-y Cumulative incidence of death (as first event), % (SE)	1.8 (0.4)	0.8 (0.3)	5.9 (2.0)	2.5 (1.8)	2.4 (2.4)	<.001^b^
**Relapse cohort**						
No.	544	366	88	71	19	NA
5-y OS from date of first relapse, % (SE)	22.9 (2.2)	26.5 (2.9)	16.3 (4.1)	18.9 (6.0)	0.0 (0.0)	.04^c^
Subsequent enrollment in COG groupwide early phase trial, No. (%)	44 (8.1)	32 (8.7)	5 (5.7)	7 (9.9)	0	.41
Subsequent enrollment in COG limited site early phase trial, No. (%)	46 (8.5)	34 (9.3)	7 (8.0)	4 (5.6)	1 (5.3)	.72

Abbreviations: COG, Children’s Oncology Group; NA, not applicable; OS, overall survival.

^a^ Kruskall-Wallis test.

^b^ Gray test.

^c^ Log-rank test.

#### Early Trial Withdrawal

Among patients on ANBL0532, 37.1% (184 of 496) withdrew early for reasons other than progression during induction, with no significant differences by race and ethnicity (Table 3). Among patients in ANBL09P1 and ANBL12P1, 37.0% (74 of 200) discontinued participation during induction for reasons other than progression, with no significant differences by race and ethnicity.

#### Relapse and Progression

Among patients enrolled on induction/consolidation trials, the 5-year cumulative incidence of relapse (SE) as first event was 48.5% (1.9%) with no significant difference by race and ethnicity (Table 3). Among patients enrolled on post-consolidation trials, the 5-year cumulative incidence of relapse (SE) as first event was 37.5% (1.6%) with no significant difference by race and ethnicity.

#### Death

Among patients enrolled on induction/consolidation trials, the 5-year cumulative incidence of death (SE) as first event significantly differed by race and ethnicity: 5.9% (1.1%) for White patients, 4.6% (2.0%) for Black patients, 9.5% (3.5%) for Hispanic patients, and 16.2% (7.7%) for patients of other race (*P* = .049) (Table 3).

Among patients enrolled in post-consolidation trials, the 5-year cumulative incidence of death (SE) as first event differed significantly by race and ethnicity: 0.8% (0.3%) for White patients, 5.9% (2.0%) for Black patients, 2.5% (1.8%) for Hispanic patients, and 2.4% (2.4%) for patients of other race (*P* = .001) (Table 3).

### Relapse Cohort Characteristics

Of the 544 patients enrolled on induction/consolidation trials and/or post-consolidation trials who relapsed, 88 (16.2%) were Black, 71 (13.1%) were Hispanic, 19 (3.5%) were of other race, and 366 (67.3%) were White (eTable 8 in Supplement 1). Race and ethnicity proportions were similar to those of the overall frontline induction/consolidation and post-consolidation cohorts (Table 1; eTable 8 in Supplement 1). Among patients with relapsed or refractory disease, there were no differences in age, sex, *MYCN* amplification, tumor histology, or INSS stage by race and ethnicity (eTable 8 in Supplement 1).

### Survival After Relapse

The median follow-up for the 126 patients still alive was 7.1 years (IQR, 5.6-9.5 years). OS from time of first relapse differed significantly by race and ethnicity (*P* = 0.04) (Table 3; Figure 2E). Five-year OS (SE) among patients of other race (0.0% [0.0%]) was inferior compared with OS among Black (16.3% [4.1%]), Hispanic (18.9% [6.0%]), and White patients (26.5% [2.9%]).

### Enrollment on Early Phase Trials After Relapse

Among the 544 patients who relapsed after frontline trial enrollment, 8.1% (n = 44) subsequently enrolled on COG groupwide early phase trials, with no significant difference by race and ethnicity. In addition, 8.5% (n = 46) enrolled in COG limited institution early phase trials, with no significant difference by race and ethnicity.

## Discussion

Children of racially and ethnically marginalized groups with high-risk neuroblastoma—those identified as Black or Hispanic—experienced inferior OS compared with White children despite accessing uniform planned treatment on frontline COG clinical trials; differences were pronounced in the post-consolidation setting. In the induction/consolidation cohort (n = 696) and the post-consolidation cohort (n = 935), race and ethnicity was independently associated with OS after adjusting for disease-associated factors. To our knowledge, this is the first study to comprehensively evaluate survival outcome disparities in a clinical trial cohort of children with high-risk neuroblastoma and to investigate potential care delivery mechanisms associated with these disparities.

Compared with White children, Hispanic children had worse OS after enrollment on induction/consolidation trials, and both Hispanic and Black children had worse OS after enrollment on post-consolidation trials. These findings mirror published population-based data indicating that Hispanic and Black children experienced inferior OS after treatment on post-consolidation immunotherapy trials.^36^ Children in our cohort categorized as other race did not experience inferior OS on induction/consolidation or post-consolidation trials. This finding differs from prior work identifying inferior OS for Asian and Native American children enrolled on the ANBL00B1 biology study,^3^ and suggests that frontline trial enrollment may mitigate disparities for these populations, and/or that this group’s small sample size and heterogeneity were associated with differences across studies. Larger and disaggregated analyses of diverse population groups are needed. Taken together, these data suggest that in the contemporary era of immunotherapy, Hispanic and Black children face worse outcomes compared with White children.

Our data did not recapitulate previously published findings of socioeconomic disparities in EFS and OS among children receiving post-consolidation immunotherapy.^6^ Differences in analytic design may explain this discrepancy. Given the intersectionality of race and ethnicity and SES due to structural racism, further analyses to investigate the compounding associations of these factors are warranted. Work investigating socioeconomic disparities by parent-reported poverty measures is ongoing (NCT03126916) and can elucidate this association.

National calls for equity in oncology have focused on trial access.^37^ However, our data demonstrate that members of marginalized racial and ethnic groups face disparate OS even after accessing clinical trials. We used trial-collected data to evaluate a priori hypotheses that early trial discontinuation, delays in induction therapy, or increased rates of relapse may explain worse survival among members of marginalized racial and ethnic groups. We did not identify significant differences by race and ethnicity when evaluating any of these mechanisms.

The lack of difference in relapse as first event by race and ethnicity in both the induction/consolidation trials and post-consolidation trials suggests that disparate death from toxicities or postrelapse care may underlie survival inequities. We specifically explored early phase trial enrollment as a proxy for access to life-extending care. In contrast to data from adults^38^ and a population-based pediatric study suggesting disparate early phase trial participation,^39^ we found no differences in COG early phase trial enrollment by race and ethnicity. We separately examined limited institution and groupwide early phase trial participation to evaluate the hypothesis that patients from marginalized communities may have less access to limited institution trials beyond their home center. We identified no disparities in limited institution trial enrollment by race and ethnicity, suggesting that access to trials may not be associated with inequities in postrelapse care. Only approximately 15% of the relapse cohort enrolled in early phase trials, suggesting that unmeasured differences in postrelapse care beyond COG trial participation (eg, access to relapse MIBG [iodine 131-labeled metaiodobenzylguanidine] therapies, radiotherapy, immunotherapies, or other consortia early phase trials) may underlie observed disparities.

We explored whether patients experienced disparities in cumulative incidence of death as first event as a proxy for toxicities. We identified significantly higher rates of death as first event among Hispanic patients and patients of other race in the induction/consolidation cohort. In the post-consolidation cohort, Black patients, Hispanic patients, and patients of other race also experienced higher rates of death as first event compared with White patients. These data warrant systematic investigation into potential differential toxicities experienced by race and ethnicity among patients enrolled in frontline trials.

### Limitations

There are several limitations to this study. We lacked specificity in how race and ethnicity were collected in the trials and how multiracial patients were categorized. In addition, we were missing race and ethnicity, insurance, or zip code for 810 patients. It is possible that data may not have been missing at random, and missing data may have skewed results; however, disease-associated characteristics did not vary between included vs excluded patients (eTable 1 in Supplement 1). This is an exploratory, hypothesis-generating study in which we used backward elimination in our multivariable modeling approach. Future studies should confirm these findings using other approaches in the context of testing specific hypotheses. Although we captured clinical trial enrollment and discontinuation, we were unable to capture receipt of planned therapies, toxicities, and delays. Furthermore, we were unable to assess data regarding enrollment in early phase trials outside of COG. Relative sample sizes did not allow disaggregation of the other race category, further marginalizing patients by limiting our ability to parse the implications of observed inferior outcomes for specific populations. Finally, race and ethnicity are social constructs that do not explain disparate outcomes by themselves. Identifying the experience of adverse social determinants of health, structural barriers that disproportionately affect these communities, and potential toxicity associations with genetic ancestry across groups will provide further insight into factors associated with disparities. Children’s Oncology Group neuroblastoma studies are collecting comprehensive social determinants of health data, and future analyses will focus on these factors.

## Conclusions

Our data add to the existing literature demonstrating survival disparities among children with cancer in 2 important ways. First, in a large trial-based cohort of children with high-risk neuroblastoma, we demonstrate that access to clinical trials alone is inadequate to mitigate survival disparities. Furthermore, we found that racially and ethnically and socioeconomically marginalized children, unlike adults, appear to have equitable access to early phase COG trials. Second, we highlight ways in which trial-collected data can be leveraged to identify or disaffirm mechanisms associated with disparities. In our cohort, hypotheses that marginalized children would experience higher rates of trial discontinuation or delays were not supported.

These findings should refocus pediatric oncology equity efforts toward understanding why survival disparities occur despite participation in trials (eg, toxicities, therapeutic resistance, differences in supportive care or postrelapse therapies). Given that children of marginalized racial and ethnic groups with neuroblastoma access clinical trials equitably, these findings reinforce the urgent need for trial-embedded health equity interventions to support marginalized communities while concurrently evaluating biological, social, and structural mechanisms associated with disparities.

## Notes

Supplement 1. **eTable 1.** Characteristics of Included Patients vs Excluded Patients **eTable 2.** Frontline Children’s Oncology Group Trial With Primary Aims and Findings **eTable 3.** Children’s Oncology Group Early Phase Trials Included in Analyses **eMethods/eResults.** Exploratory Analyses by Socioeconomic Status Measures **eTable 4.** Characteristics of Induction/Consolidation Study Patients by Socioeconomic Status Measures (ANBL0532, ANBL09P1, ANBL12P1) **eTable 5.** Characteristics of Post-Consolidation Study Patients by Socioeconomic Status (ANBL0032 and ANBL0931) **eTable 6.** Potential Mechanisms for Disparate Survival by Socioeconomic Measures **eTable 7.** Characteristics of Patients With Relapsed or Progressive Disease by Socioeconomic Status **eFigure 1.** Survival Curves for Induction/Consolidation, Post-Consolidation, and Relapse Cohorts by Household Poverty **eFigure 2.** Survival Curves for Induction/Consolidation, Post-Consolidation, and Relapse Cohorts by Area Poverty **eFigure 3.** Survival Curves for Induction/Consolidation, Post-Consolidation, and Relapse Cohorts by Rurality **eTable 8.** Characteristics of Patients With Relapsed or Progressive Disease by Race and Ethnicity

Supplement 2.
Data Sharing Statement
