# Supplementary material for: Racial and Ethnic Survival Disparities Among Children With High-Risk Neuroblastoma: A Children’s Oncology Group Report
Source: JAMA Netw Open. 2025 Feb 14;8(2):e2458531. doi: 10.1001/jamanetworkopen.2024.58531 (PMC11829236; doi:10.1001/jamanetworkopen.2024.58531)
Supplement: Supplement 1. — eTable 1. Characteristics of Included Patients vs Excluded Patients eTable 2. Frontline Children’s Oncology Group Trial With Primary Aims and Findings eTable 3. Children’s Oncology Group Early Phase Trials Included in Analyses eMethods/eResults. Exploratory Analyses by Socioeconomic Status Measures eTable 4. Characteristics of Induction/Consolidation Study Patients by Socioeconomic Status Measures (ANBL0532, ANBL09P1, ANBL12P1) eTable 5. Characteristics of Post-Consolidation Study Patients by Socioeconomic Status (ANBL0032 and ANBL0931) eTable 6. Potential Mechanisms for Disparate Survival by Socioeconomic Measures eTable 7. Characteristics of Patients With Relapsed or Progressive Disease by Socioeconomic Status eFigure 1. Survival Curves for Induction/Consolidation, Post-Consolidation, and Relapse Cohorts by Household Poverty eFigure 2. Survival Curves for Induction/Consolidation, Post-Consolidation, and Relapse Cohorts by Area Poverty eFigure 3. Survival Curves for Induction/Consolidation, Post-Consolidation, and Relapse Cohorts by Rurality eTable 8. Characteristics of Patients With Relapsed or Progressive Disease by Race and Ethnicity [file jamanetwopen-e2458531-s001.pdf]

Supplementary Online Content

Umaretiya PJ, Naranjo A, Zhang FF, et al. Racial and ethnic survival disparities among children with high-risk neuroblastoma: a Children's Oncology Group report. *JAMA Netw Open.* 2025;8(2):e2458531. doi:10.1001/jamanetworkopen.2024.58531

eTable 1. Characteristics of Included Patients vs Excluded Patients
eTable 2. Frontline Children's Oncology Group Trial With Primary Aims and Findings
eTable 3. Children's Oncology Group Early Phase Trials Included in Analyses
eMethods/eResults. Exploratory Analyses by Socioeconomic Status Measures
eTable 4. Characteristics of Induction/Consolidation Study Patients by Socioeconomic Status Measures (ANBL0532, ANBL09P1, ANBL12P1)
eTable 5. Characteristics of Post-Consolidation Study Patients by Socioeconomic Status (ANBL0032 and ANBL0931)
eTable 6. Potential Mechanisms for Disparate Survival by Socioeconomic Measures
eTable 7. Characteristics of Patients With Relapsed or Progressive Disease by Socioeconomic Status
eFigure 1. Survival Curves for Induction/Consolidation, Post-Consolidation, and Relapse Cohorts by Household Poverty
eFigure 2. Survival Curves for Induction/Consolidation, Post-Consolidation, and Relapse Cohorts by Area Poverty
eFigure 3. Survival Curves for Induction/Consolidation, Post-Consolidation, and Relapse Cohorts by Rurality
eTable 8. Characteristics of Patients With Relapsed or Progressive Disease by Race and Ethnicity

This supplementary material has been provided by the authors to give readers additional information about their work.

© 2025 Umaretiya PJ et al. *JAMA Network Open.*

**eTable 1.** Characteristics of Included Patients vs Excluded Patients			
**Induction/Consolidation Cohort**			
	Included in Induction/ Consolidation Cohort N=696	Excluded N=200	p-value*
Age, N(%)			
< 18 mos	86 (12.4)	21 (10.5)	0.4755
≥18 mos	610 (87.6)	179 (89.5)	
Sex, N(%)			
Male	404 (58.1)	110 (55.0)	0.4427
Female	292 (42.0)	90 (45.0)	
Tumor *MYCN* status, N(%)			
Amplified	272 (43.3)	59 (35.5)	
Not amplified	356 (56.7)	107 (64.5)	0.0710
Missing	68	34	
Tumor histology, N(%)			
Unfavorable	603 (96.3)	156 (94.0)	
Favorable	23 (3.7)	10 (6.0)	0.1780
Missing	70	34	
INSS stage, N(%)			
1	1 (0.1)	1 (0.5)	
2A	2 (0.3)	2 (1.0)	
2B	6 (0.9)	3 (1.5)	0.7610[a]
3	71 (10.2)	15 (7.5)	
4	614 (88.2)	178 (89.0)	
4S	2 (0.3)	1 (0.5)	
EOI disease response, N(%)[b]			
CR	113 (18.5)	32 (17.8)	
VGPR	148 (24.2)	49 (27.2)	
PR	207 (33.8)	68 (37.8)	
MR	56 (9.2)	12 (6.7)	0.0730[c]
NR	33 (5.4)	10 (5.6)	
PD	55 (9.0)	9 (5.0)	
Missing	84	20	
Trial, N(%)			
ANBL0532	496 (71.3)	156 (78.0)	
ANBL09P1 (limited)	82 (11.8)	16 (8.0)	0.1472
ANBL12P1	118 (17.0)	28 (14.0)	
**Post-Consolidation Cohort**			
	Included in post-Consolidation Cohort N=935	Excluded N=610	p-value*
Age, N(%)			
< 18 mos	145 (15.5)	87 (14.3)	0.5029
≥18 mos	790 (84.5)	523 (85.7)	
Sex, N(%)			
Male	567 (60.6)	354 (58.0)	0.3070
Female	368 (39.4)	256 (42.0)	
Tumor *MYCN* status, N(%)			
Amplified	325 (45.7)	156 (42.2)	
Not amplified	386 (54.3)	214 (57.8)	0.2654
Missing	224	240	
Tumor histology, N(%)			
Unfavorable	657 (94.1)	332 (95.4)	

© 2025 Umaretiya PJ et al. *JAMA Network Open.*

Favorable	41 (5.9)	16 (4.6)	0.3916
Missing	237	262	
INSS stage, N(%)			
1	4 (0.5)	3 (0.7)	
2A	5 (0.6)	3 (0.7)	
2B	19 (2.4)	9 (2.0)	
3	102 (12.8)	50 (11.2)	0.2483[a]
4	657 (82.4)	379 (85.0)	
4S	10 (1.3)	2 (0.4)	
Missing	138	164	
EOI disease response, N(%)[b]			
CR	260 (29.8)	103 (24.6)	
VGPR	273 (31.3)	147 (35.1)	
PR	322 (36.9)	156 (37.2)	
MR	13 (1.5)	13 (3.1)	0.2525[c]
NR	1 (0.1)	0 (0.0)	
PD	4 (0.5)	0 (0.0)	
Missing	62	191	
Trial, N(%)			
ANBL0032	855 (91.4)	585 (95.9)	0.0007
ANBL0931	80 (8.6)	25 (4.1)	

[*]Missing values excluded from p-value comparisons
[a]Non-stage 4 vs Stage 4
[b]EOI; end-of-induction; CR; complete response; VGPR; very good partial response; PR; partial response; MR; mixed response; NR; no response; PD; progressive disease
[c]PR or better vs. less than PR (MR, NR, PD)

© 2025 Umaretiya PJ et al. *JAMA Network Open.*

**eTable 2.** Frontline Children's Oncology Group Trial With Primary Aims and Findings				
**Trial**	**Phase of therapy**	**Groupwide[a] vs. limited Institution trial[b]**	**Primary aims**	**Primary results**
ANBL0532[16]	Induction/Consolidation	Groupwide	Randomized control trial of single vs. tandem autologous stem cell transplant as consolidative therapy	Statistically significant improved survival for tandem transplant cohort
ANBL091P1[17]	Induction/Consolidation	Limited institution	Pilot evaluation of the feasibility of I-MIBG during induction therapy	Administering ^{131}I-MIBG during induction therapy was feasible and tolerable
ANBL12P1[18]	Induction/Consolidation	Groupwide	Pilot evaluation of the feasibility and tolerability of a busulfan/melphalan regimen for autologous stem cell transplant consolidative therapy	Busulfan/melphalan regimen after COG induction was tolerable
ANBL0032[19]	Post-consolidation	Groupwide	Randomized control trial of targeted immunotherapy with monoclonal antibody dinutuximab plus iroretinoin vs standard of care isoretinoin as post-consolidation	Randomization stopped early after a statistically significant improvement in EFS among patients assigned to the immunotherapy arm
ANBL0931[20]	Post-consolidation	Limited institution	Pilot evaluation of the safety and toxicity of dinutixumab immunotherapy	Dinutuximab had significant but manageable treatment-related toxicities

[a]Groupwide = available at ~180 COG centers
[b]Limited institution = available at ~20 COG centers

© 2025 Umaretiya PJ et al. *JAMA Network Open.*

**eTable 3.** Children's Oncology Group Early Phase Trials Included in Analyses	
Groupwide early phase trials[a]	ANBL1221, ANBL1021, ANBL0421, ADVL0524, ADVL0421, ADVL0524, ADVL0525, ADVL0821, ADVL0921, ADVL1522
Limited institution early phase trials[b]	ANBL0322, ANBL0621, ADVL0812, ADVL0912, ADVL1322, ADVL0413, ADVL0414, ADVL0416, ADVL0419, ADVL0517, ADVL0612, ADVL0712, ADVL0714, ADVL0813, ADVL0815, ADVL0816, ADVL0911, ADVL0916, ADVL0918, ADVL0919, ADVL1011, ADVL1013, ADVL1014, ADVL1111, ADVL1112, ADVL1115, ADVL1211, ADVL1212, ADVL1213, ADVL1312, ADVL1314, ADVL1315, ADVL1411, ADVL1412, ADVL1414, ADVL1416, ADVL1513, ADVL1514, ADVL1515, ADVL1614, ADVL1615

© 2025 Umaretiya PJ et al. *JAMA Network Open.*

eMethods/eResults. Exploratory Analyses by Socioeconomic Status Measures

Statistical analysis
Patient demographics including race and ethnicity, tumor *MYCN* status, tumor histology, INSS stage, and EOI disease response were summarized descriptively by socioeconomic measures. Patient demographic and disease-associated characteristics were compared by socioeconomic status measures using a chi-squared test or Fisher's exact test for categorical variables and a Wilcoxon rank-sum test for continuous variables. OS and EFS curves were plotted using Kaplan-Meier methods and EFS and OS were estimated with standard errors.[26] Associations between socioeconomic status (SES) measures and survival outcomes were evaluated with univariate Cox proportional models. Multivariable Cox models of survival outcomes initially included all statistically significant univariate factors ($p<0.05$) and used a backward selection process.

Care delivery inflection points including trial discontinuation for non-progression, induction delays, and relapse were summarized descriptively and compared by socioeconomic status measures using a chi-squared or Fisher's exact test for categorical variables and a Wilcoxon rank-sum test for continuous variables.

Associations between socioeconomic status measures and early phase trial enrollment for patients who relapse were evaluated with univariate Cox proportional models. Multivariable models compared early phase trial enrollment by race and ethnicity adjusted for significant univariate factors ($p<0.05$) using a Wald test.

Patient characteristics in upfront trials
Induction/Consolidation cohort (ANBL0532, ANBL09P1, ANBL12P1)
The induction/consolidation cohort included 696 patients treated on upfront induction/consolidation trials (Table 1). In this cohort, 32.9% (n=229) had household-level poverty exposure, 25.9% (n=180) had area-level poverty exposure, and 15.2% had rural exposure. There were no differences in age, sex, *MYCN* amplification, tumor histology, or INSS stage by SES measures.

Post-Consolidation cohort (ANBL0032 and ANBL0931)
The post-consolidation cohort included 935 patients (Table 2) of whom 373 were included in the Induction/Consolidation cohort. In this cohort, 30.8% (n=288) had household-level poverty exposure, 23.9% (n=223) had area-level poverty exposure, and 15.3% (n=143) had rural exposure. There were no differences in age, sex, *MYCN* amplification, tumor histology, INSS stage, or EOI response by socioeconomic status measures.

Associations between socioeconomic status and survival on upfront clinical trials

Survival analyses for Induction/Consolidation cohort
In univariate analyses, EFS did not differ by household-level poverty, area-level poverty, or rurality.

In univariate analyses for OS, patients with household-level poverty experienced significantly inferior OS (P=0.0371) compared to those without household-level poverty exposure (52.9±3.6% vs. 62.8±2.4% at 5-years; HR=1.28. 95% CI=1.01-1.62). OS did not differ by area-level poverty or rurality. In multivariable analyses of OS, SES measures did not remain significantly associated with survival after adjusting for race and ethnicity and disease-associated factors.

Survival analyses for Post-Consolidation cohort
In univariate analyses, EFS did not differ by household-level poverty, area-level poverty, or rurality.

In univariate analyses for OS, patients with household-level poverty experienced significantly inferior OS (P=0.0221) compared to those without household-level poverty exposure (68.1±3.0% vs. 75.1±1.8% at 5-years; HR=1.32. 95% CI=1.04-1.69). OS did not differ by area-level poverty or rurality. In multivariable analyses of OS, SES measures did not remain significantly associated with survival after adjusting for race and ethnicity and disease-associated factors.

Exploration of care delivery mechanisms underlying survival outcome disparities

© 2025 Umaretiya PJ et al. *JAMA Network Open.*

Three care delivery inflection points were *a priori* designated for evaluation of disparate survival outcomes by socioeconomic status: delays during induction chemotherapy, early trial discontinuation, and frequency of relapsed disease.

Delays during induction chemotherapy
Overall median length of induction for patients enrolled on ANBL0532 was 168 days (interquartile range 155,179) and there were no significant differences by socioeconomic status measures (Table 3).

Early trial discontinuation
Among patients enrolled on ANBL0532, 37.1% of patients discontinued participation for reasons other than progression during induction with no significant differences by socioeconomic status measures. Among patients enrolled on ANBL09P1 and ANBL12P1, 37.0% of patients discontinued participation during induction for reasons other than progression with no significant differences by socioeconomic status measures.

Relapsed disease

Induction/Consolidation cohort
Among patients enrolled on induction/consolidation trials, the 5-year cumulative incidence of relapse as a first event was 48.5±1.9% with no differences by socioeconomic status measures.

Post-Consolidation cohort
Among patients enrolled on post-consolidation trials, the 5-year cumulative incidence of relapse as a first event was 37.5±1.6% with no differences by socioeconomic status measures.

Death
Induction/Consolidation cohort
There was no difference in the cumulative incidence of death as a first event by socioeconomic status measures.

Post-Consolidation cohort
Patients exposed to area-level poverty had a higher cumulative incidence rate of death (3.8±1.3% at 5-years) compared to those without area-level poverty (1.2±0.4% at 5 years; *P*=0.0243). There was no difference in the cumulative incidence of death as a first event by household-level poverty or rurality.

Relapse cohort characteristics
Socioeconomic characteristics of the 544 patients enrolled on induction/consolidation trials and/or post-consolidation trials who experienced relapse included 33.6% (n=183) with household-level poverty exposure, 25.9% (n=141) with area-level poverty exposure, and 15.4% (n=84) with rural exposure (Table 4). Proportions of patients by socioeconomic measures in the relapse cohort were similar to those of the overall cohort enrolled on upfront induction/consolidation and post-consolidation studies. Among patients with relapsed disease, there were no differences in age, *MYCN* amplification, tumor histology, or INSS stage by socioeconomic status (Table 4).

Survival after relapsed disease
Patients exposed to household-level poverty had inferior OS after first relapse (18.5±3.6% at 5-years) compared to those unexposed (25.1±2.8% at 5-years, *P*=0.0072). There was no difference in OS by area-level poverty or rurality.

Enrollment to early phase trials after relapse
Enrollment on early phase trials after relapse was evaluated by socioeconomic status. Early phase trials were categorized as groupwide (available at all COG institutions nationwide) or limited (available at a subset of COG institutions).

Among the 544 patients who experienced relapse after enrollment on an upfront trial, 8.1% (n=44) patients subsequently enrolled on COG groupwide early phase trials with no significant difference by socioeconomic status measures. In addition, 8.5% (n=46) enrolled on COG limited institution early phase trials with no significant difference by socioeconomic status measures.

© 2025 Umaretiya PJ et al. *JAMA Network Open.*

eTable 4. Characteristics of Induction/Consolidation Study Patients by Socioeconomic Status Measures (ANBL0532, ANBL09P1, ANBL12P1)

	Overall N=696	No household poverty N=467	Household poverty N=229	p-value	No Area poverty N=513	Area poverty N=180	Unknown area poverty N=3	p-value (among known)	Urban N=589	Rural N=106	Unknown N=1	p-value (among known)
Sociodemographic characteristics												
Age, N(%)				0.9422				0.3805				0.7771
< 18 mos	86 (12.4)	58 (12.4)	28 (12.2)		67 (13.1)	19 (10.6)	0 (0.0)		72 (12.2)	14 (13.2)	0 (0.0)	
≥18 mos	610 (87.6)	409 (87.6)	201 (87.8)		446 (86.9)	161 (89.4)	3 (100.0)		517 (87.8)	92 (86.8)	1 (100.0)	
Sex, N(%)				0.7345				0.0925				0.3323
Male	404 (58.1)	269 (57.6)	135 (59.0)		288 (56.1)	114 (63.3)	2 (66.7)		337 (57.2)	66 (62.3)	1 (100.0)	
Female	292 (42.0)	198 (42.4)	94 (41.1)		225 (43.9)	66 (36.7)	1 (33.3)		252 (42.8)	40 (37.7)	0 (0.0)	
Race/Ethnicity				<0.0001				<0.0001				0.0038
White	481 (69.1)	360 (77.1)	121 (52.8)		390 (76.0)	88 (48.9)	3 (100.0)		392 (66.6)	88 (83.0)	1 (100.0)	
Black	109 (15.7)	54 (11.6)	55 (24.0)		53 (10.3)	56 (31.1)	0 (0.0)		97 (16.5)	12 (11.3)	0 (0.0)	
Hispanic	79 (11.4)	33 (7.1)	46 (20.1)		46 (9.0)	33 (18.3)	0 (0.0)		76 (12.9)	3 (2.8)	0 (0.0)	
Other	27 (3.9)	20 (4.3)	7 (3.1)		24 (4.7)	3 (1.7)			24 (4.1)	3 (2.8)	0 (0.0)	
Disease characteristics												
Tumor MYCN status, N (%)				0.7043				0.1219				0.7275
Amplified	272 (43.3)	181 (42.8)	91 (44.4)		194 (41.7)	78 (48.8)	0 (0.0)		231 (43.1)	41 (45.1)	0 (0.0)	
Not amplified	356 (56.7)	242 (57.2)	114 (55.6)		271 (58.3)	82 (51.3)	3 (100.0)		305 (56.9)	50 (55.0)	1 (100.0)	
Unknown	68	44	24		48	20	0		53	15	0	
Tumor histology, N(%)				0.5182				0.1760				1.000*
Unfavorable	603 (96.3)	407 (96.0)	196 (97.0)		447 (95.7)	153 (98.1)	3 (100.0)		511 (96.2)	91 (96.8)	1 (100.0)	
Favorable	23 (3.7)	17 (4.0)	6 (3.0)		20 (4.3)	3 (1.9)	0 (0.0)		20 (3.8)	3 (3.2)	0 (0.0)	
Unknown	70	43	27		46	24	0		58	12	0	
INSS stage, N(%)				0.1346 (Stage 4 vs. Non-Stage 4)				0.3763 (Stage 4 vs. Non-Stage 4)				0.4123 (Stage 4 vs. Non-Stage 4)
1	1 (0.1)	1 (0.2)	0 (0.0)		1 (0.2)	0 (0.0)	0 (0.0)		1 (0.2)	0 (0.0)	0 (0.0)	
2A	2 (0.3)	1 (0.2)	1 (0.4)		2 (0.4)	0 (0.0)	0 (0.0)		2 (0.3)	0 (0.0)	0 (0.0)	
2B	6 (0.9)	4 (0.9)	2 (0.9)		6 (1.2)	0 (0.0)	0 (0.0)		4 (0.7)	2 (1.9)	0 (0.0)	
3	71 (10.2)	53 (11.4)	18 (7.9)		53 (10.3)	18 (10.0)	0 (0.0)		63 (10.7)	8 (7.6)	0 (0.0)	
4S	2 (0.3)	2 (0.4)	0 (0.0)		2 (0.4)	0 (0.0)	0 (0.0)		2 (0.3)	0 (0.0)	0 (0.0)	
4	614 (88.2)	406 (86.9)	208 (90.8)		449 (87.5)	162 (90.0)	3 (100.0)		517 (87.8)	96 (90.6)	1 (100.0)	
EOI disease response, N(%)				0.0854 (CR/VGPR/ PR vs. MR/ NR/PD)				0.0584 (CR/VGPR/ PR vs. MR/ NR/PD)				0.5062 (CR/VGPR/ PR vs. MR/ NR/PD)
CR	113 (18.5)	77 (19.0)	36 (17.5)		83 (18.7)	29 (17.7)	1 (33.3)		96 (18.6)	17 (17.7)	0 (0.0)	
VGPR	148 (24.2)	96 (23.7)	52 (25.2)		101 (22.7)	47 (28.7)	0 (0.0)		119 (23.1)	29 (30.2)	0 (0.0)	
PR	207 (33.8)	146 (36.0)	61 (29.6)		166 (37.3)	41 (25.0)	0 (0.0)		182 (35.3)	25 (26.0)	0 (0.0)	
MR	56 (9.2)	35 (8.6)	21 (10.2)		37 (8.3)	18 (11.0)	1 (33.3)		44 (8.5)	11 (11.5)	1 (100.0)	
NR	33 (5.4)	21 (5.2)	12 (5.8)		19 (4.3)	13 (7.9)	1 (33.3)		27 (5.2)	6 (6.3)	0 (0.0)	
PD	55 (9.0)	31 (7.6)	24 (11.7)		39 (8.8)	16 (9.8)	0 (0.0)		47 (9.1)	8 (8.3)	0 (0.0)	
Missing	84	61	23		68	16	0		74	10	0	

© 2025 Umaretiya PJ et al. *JAMA Network Open.*

Trial, N(%)				0.1534				0.1162				0.1157
ANBL0532	496 (71.3)	331 (70.9)	165 (72.1)		362 (70.6)	131 (72.8)	3 (100.0)		421 (71.5)	74 (69.8)	1 (100.0)	
ANBL09P1 (limited)	82 (11.8)	62 (13.3)	20 (8.7)		68 (13.3)	14 (7.8)	0 (0.0)		74 (12.6)	8 (7.6)	0 (0.0)	
ANBL12P1	118 (17.0)	74 (15.9)	44 (19.2)		83 (16.2)	35 (19.4)	0 (0.0)		94 (16.0)	24 (22.6)	0 (0.0)	

© 2025 Umaretiya PJ et al. *JAMA Network Open.*

eTable 5. Characteristics of Post-Consolidation Study Patients by Socioeconomic Status (ANBL0032 and ANBL0931)

	Overall N=935	No household poverty N=647	Household poverty N=288	p-value (among known)[a]	No area poverty N=707	Area poverty N=223	Unknown area poverty N=5	p-value (among known)[a]	Urban N=791	Rural N=143	Unknown N=1	p-value (among known)[a]
Sociodemographic characteristics												
Age, N(%)				0.1028				0.8707				0.9599
< 18 mos	145 (15.5)	92 (14.2)	53 (18.4)		111 (15.7)	34 (15.2)	0 (0.0)		123 (15.5)	22 (15.4)	0 (0.0)	
≥18 mos	790 (84.5)	555 (85.8)	235 (81.6)		596 (84.3)	189 (84.7)	5 (100.0)		668 (84.5)	121 (84.6)	1 (100.0)	
Sex, N(%)				0.3351				0.5295				0.5342
Male	567 (60.6)	399 (61.7)	168 (58.3)		424 (60.0)	139 (62.3)	4 (80.0)		476 (60.2)	90 (62.9)	1 (100.0)	
Female	368 (39.4)	248 (38.3)	120 (41.7)		283 (40.0)	84 (37.7)	1 (20.0)		315 (39.8)	53 (37.1)	0 (0.0)	
Race/Ethnicity				<0.0001				<0.0001				0.0429
White	662 (70.8)	510 (78.8)	152 (52.8)		548 (77.5)	111 (49.8)	3 (60.0)		547 (69.2)	115 (80.4)	0 (0.0)	
Black	145 (15.5)	74 (11.4)	71 (24.7)		74 (10.5)	70 (31.4)	1 (20.0)		131 (16.6)	14 (9.8)	0 (0.0)	
Hispanic	87 (9.3)	37 (5.7)	50 (17.4)		52 (7.4)	34 (15.2)	1 (20.0)		78 (9.9)	8 (5.6)	1 (100.0)	
Other	41 (4.4)	26 (4.0)	15 (5.2)		33 (4.7)	8 (3.6)	0 (0.0)		35 (4.4)	6 (4.2)	0 (0.0)	
Disease characteristics												
Tumor MYCN status, N (%)				0.3050				0.1435				0.2968
Amplified	325 (45.7)	220 (44.4)	105 (48.6)		238 (44.2)	85 (50.6)	2 (50.0)		281 (46.4)	43 (41.0)	1 (100.0)	
Not amplified	386 (54.3)	275 (55.6)	111 (51.4)		301 (55.8)	83 (49.4)	2 (50.0)		324 (53.6)	62 (59.0)	0 (0.0)	
Unknown	224	152	72		168	55	1		186	38	0	
Tumor histology, N(%)				0.8807				0.2128				0.5797
Unfavorable	657 (94.1)	456 (94.2)	201 (93.9)		500 (93.6)	154 (96.3)	3 (75.0)		555 (93.9)	101 (95.3)	1 (100.0)	
Favorable	41 (5.9)	28 (5.8)	13 (6.1)		34 (6.4)	6 (3.8)	1 (25.0)		36 (6.1)	5 (4.7)	0 (0.0)	
Unknown	237	163	74		173	63	1		200	37	0	
INSS stage, N(%)				0.3435 (Stage 4 vs. Non-Stage 4)				0.4394 (Stage 4 vs. Non-Stage 4)				0.9781 (Stage 4 vs. Non-Stage 4)
1	4 (0.5)	4 (0.7)	0 (0.0)		4 (0.7)	0 (0.0)	0 (0.0)		4 (0.6)	0 (0.0)	0 (0.0)	
2A	5 (0.6)	4 (0.7)	1 (0.4)		5 (0.8)	0 (0.0)	0 (0.0)		5 (0.7)	0 (0.0)	0 (0.0)	
2B	19 (2.4)	14 (2.5)	5 (2.1)		16 (2.7)	3 (1.6)	0 (0.0)		15 (2.2)	4 (3.3)	0 (0.0)	
3	102 (12.8)	73 (13.2)	29 (11.9)		78 (12.9)	24 (12.6)	0 (0.0)		88 (13.0)	14 (11.7)	0 (0.0)	
4S	10 (1.3)	7 (1.3)	3 (1.2)		7 (1.2)	3 (1.6)	0 (0.0)		7 (1.0)	3 (2.5)	0 (0.0)	
4	657 (82.4)	452 (81.6)	205 (84.4)		493 (81.8)	160 (84.2)	4 (100.0)		557 (82.4)	99 (82.5)	1 (100.0)	
Unknown	138	93	45		104	33	1		115	23	0	
EOI disease response, N(%)				0.8910 (CR/VGPR/ PR vs. MR/ NR/PD)				0.7807* (CR/VGPR/ PR vs. MR/ NR/PD)				0.1743* (CR/VGPR/ PR vs. MR/ NR/PD)
CR	260 (29.8)	177 (29.7)	83 (29.9)		198 (30.0)	61 (29.0)	1 (33.3)		222 (30.0)	38 (28.6)	0 (0.0)	
VGPR	273 (31.3)	193 (32.4)	80 (28.8)		201 (30.5)	70 (33.3)	2 (66.7)		227 (30.7)	45 (33.8)	1 (100.0)	
PR	322 (36.9)	213 (35.8)	109 (39.2)		248 (37.6)	74 (35.2)	0 (0.0)		277 (37.5)	45 (33.8)	0 (0.0)	
MR	13 (1.5)	9 (1.5)	4 (1.4)		9 (1.4)	4 (1.9)	0 (0.0)		10 (1.4)	3 (2.3)	0 (0.0)	
NR	1 (0.1)	1 (0.2)	0 (0.0)		1 (0.2)	0 (0.0)	0 (0.0)		1 (0.1)	0 (0.0)	0 (0.0)	
PD	4 (0.5)	2 (0.3)	2 (0.7)		3 (0.5)	1 (0.5)	0 (0.0)		2 (0.3)	2 (1.5)	0 (0.0)	
Missing	62	52	10		47	13	2		52	10	0	
Trial, N(%)				<0.0001 (ANBL0032 Pre-2009 vs. ANBL0032 Post-2009 vs. ANBL0931)				0.3139 (ANBL0032 Pre-2009 vs. ANBL0032 Post-2009 vs. ANBL0931)				0.5705 (ANBL0032 Pre-2009 vs. ANBL0032 Post-2009 vs.
ANBL0032												
Pre-2009 (randomization)	62 (6.6)	52 (8.0)	10 (3.5)		47 (6.6)	13 (5.8)	2 (40.0)		52 (6.6)	10 (7.0)	0 (0.0)	
Immunotherapy	33 (3.5)	31 (4.8)	2 (0.7)		24 (3.4)	9 (4.0)	0 (0.0)		28 (3.5)	5 (3.5)	0 (0.0)	
Cis-RA	29 (3.1)	21 (3.2)	8 (2.8)		23 (3.3)	4 (1.8)	2 (40.0)		24 (3.0)	5 (3.5)	0 (0.0)	
Post-2009	793 (84.8)	525 (81.1)	268 (93.1)		594 (84.0)	196 (87.9)	3 (60.0)		668 (84.5)	124 (86.7)	1 (100.0)	
ANBL0931 (limited)	80 (8.6)	70 (10.8)	10 (3.5)		66 (9.3)	14 (6.3)	0 (0.0)		71 (9.0)	9 (6.3)	0 (0.0)	

© 2025 Umaretiya PJ et al. *JAMA Network Open.*

												ANBL0931)

[a] * Indicates Fisher's Exact Test was used

© 2025 Umaretiya PJ et al. *JAMA Network Open.*

**eTable 6.** Potential Mechanisms for Disparate Survival by Socioeconomic Measures										
Induction/Consolidation cohort										
	Overall N=696 [b]	No household poverty N=467	Household poverty N=229	p-value [a]	No area poverty N=513	Area poverty N=180	p-value [a]	Urban N=589	Rural N=106	p-value [a]
Trial discontinuation for non-progression during induction (ANBL0532), N(%)	184 (37.1)	123 (37.2)	61 (37.0)	0.9670	132 (36.5)	50 (38.2)	0.7291	159 (37.8)	24 (32.4)	0.3806
Trial discontinuation for non-progression during induction (ANBL09P1, ANBL12P1), N(%)	74 (37.0)	55 (40.4)	19 (29.7)	0.1417	56 (37.1)	18 (36.7)	0.9647	64 (38.1)	10 (31.3)	0.4623
Induction delays measured by total induction length (ANBL0532) (median, IQR)	N=479 168 (155,179)	N=320 167 (154,179)	N=159 169 (156,182)	0.1131**	N=351 168 (155,179)	N=125 168 (156,179)	0.8194**	N=410 167 (155,179)	N=68 171 (155.5,181)	0.4462**
5-yr cumulative incidence of relapse (as first event) ± standard error (SE)	48.5 ± 1.9%	47.3 ± 2.4%	50.9 ± 3.4%	0.2182****	47.5 ± 2.3%	51.1 ± 3.8%	0.1164****	48.5 ± 2.1%	48.0 ± 5.0%	0.6805****
5-year cumulative incidence of death (as first event) ± SE	6.5 ± 0.9%	6.1 ± 1.1%	7.2 ± 1.7%	0.8489****	6.6 ± 1.1%	6.3 ± 1.8%	0.8255****	6.4 ± 1.0%	6.6 ± 2.4%	0.7448****
Post-Consolidation cohort										
	Overall N=935 [c]	No household poverty N=647	Household poverty N=288	p-value [a]	No area poverty N=707	Area poverty N=223	p-value[a]	Urban N=791	Rural N=143	p-value [a]
Relapse or progression (as first event), N(%)	350 (37.4)	236 (36.5)	114 (39.6)	0.3647	265 (37.5)	83 (37.2)	0.9437	295 (37.3)	55 (38.5)	0.7908
5-year cumulative incidence of relapse (as first event) ± SE	37.5 ± 1.6%	36.4 ± 1.9%	40.1 ± 2.9%	0.3404****	37.3 ± 1.8%	38.1 ± 3.3%	0.9999****	37.4 ± 1.7%	38.4 ± 4.1%	0.7814****
5-year cumulative incidence of death (as first event) ± SE	1.8 ± 0.4%	1.1 ± 0.4%	3.2 ± 1.1%	0.0517****	1.2 ± 0.4%	3.8 ± 1.3%	0.0243****	1.8 ± 0.5%	1.4 ± 1.0%	0.5767****
Relapse cohort										
	Overall N=544 [d]	No household poverty N=361	Household poverty N=183	p-value [a]	No area poverty N=399	Area poverty N=141	p-value [a]	Urban N=459	Rural N=84	p-value [a]
5-yr OS ± SE from date of first relapse	22.9 ± 2.2%	25.1 ± 2.8%	18.5 ± 3.6%	0.0072***	24.7 ± 2.7%	18.5 ± 3.9%	0.0694***	21.4 ± 2.3%	31.7 ± 6.5%	0.0717***
Enrollment on COG groupwide early phase trial	44 (8.1)	29 (8.0)	15 (8.2)	0.9473	29 (7.3)	13 (9.2)	0.4570	35 (7.6)	8 (9.5)	0.5536
Enrollment on COG limited early phase trial	46 (8.5)	34 (9.4)	12 (6.6)	0.2572	33 (8.3)	13 (9.2)	0.7285	40 (8.7)	6 (7.1)	0.6343

[a] ** Indicates Wilcoxon Rank Sum Test was used; *** Indicates Log-rank test was used; **** indicates Gray's test was used

© 2025 Umaretiya PJ et al. *JAMA Network Open.*

[b] 3 patients missing area poverty; 1 patient missing rurality
[c] 5 patients missing area poverty; 1 patient missing rurality
[d] 4 patients missing area poverty; 1 patient missing rurality

© 2025 Umaretiya PJ et al. *JAMA Network Open.*

eTable 7. Characteristics of Patients With Relapsed or Progressive Disease by Socioeconomic Status

	Overall N=544	No household poverty N=361	Household poverty N=183	p-value [a]	No neighborhood Poverty N=399	Neighborhood poverty N=141	Unknown N=4	p-value (among known) [a]	Urban N=459	Rural N=84	Unknown N=1	p-value (among known) [a]
Sociodemographic characteristics												
Age, N(%)				0.0804				0.5065				0.6783
< 18 mos	70 (12.9)	40 (11.1)	30 (16.4)		54 (13.5)	16 (11.3)	0 (0.0)		58 (12.6)	12 (14.3)	0 (0.0)	
≥18 mos	474 (87.1)	321 (88.9)	153 (83.6)		345 (86.5)	125 (88.7)	4 (100.0)		401 (87.4)	72 (85.7)	1 (100.0)	
Sex, N(%)				0.5337				0.7857				0.0001
Male	331 (60.8)	223 (61.8)	108 (59.0)		241 (60.4)	87 (61.7)	3 (75.0)		263 (57.3)	67 (79.8)	1 (100.0)	
Female	213 (39.2)	138 (38.2)	75 (41.0)		158 (39.6)	54 (38.3)	1 (25.0)		196 (42.7)	17 (20.2)	0 (0.0)	
Race/Ethnicity				<0.0001				<0.0001				0.0447
White	366 (67.3)	276 (76.5)	90 (49.2)		294 (73.7)	69 (48.9)	3 (75.0)		299 (65.1)	66 (78.6)	1 (100.0)	
Black	88 (16.2)	42 (11.6)	46 (25.1)		46 (11.5)	41 (29.1)	1 (25.0)		79 (17.2)	9 (10.7)	0 (0.0)	
Hispanic	71 (13.1)	30 (8.3)	41 (22.4)		46 (11.5)	25 (17.7)	0 (0.0)		66 (14.4)	5 (6.0)	0 (0.0)	
Other	19 (3.5)	13 (3.6)	6 (3.3)		13 (3.3)	6 (4.3)	0 (0.0)		15 (3.3)	4 (4.8)	0 (0.0)	
Disease characteristics												
Tumor MYCN status, N (%)				0.7478				0.2505				0.6770
Amplified	189 (43.2)	128 (43.7)	61 (42.1)		132 (41.8)	57 (47.9)	0 (0.0)		162 (43.7)	27 (40.9)	0 (0.0)	
Not amplified	249 (56.8)	165 (56.3)	84 (57.9)		184 (58.2)	62 (52.1)	3 (100.0)		209 (56.3)	39 (59.1)	1 (100.0)	
Unknown	106	68	38		83	22	1		88	18	0	
Tumor histology, N(%)				0.3694*				0.7391*				0.4405*
Unfavorable	413 (96.9)	279 (97.6)	134 (95.7)		303 (96.8)	108 (98.2)	2 (66.7)		348 (97.2)	64 (95.5)	1 (100.0)	
Favorable	13 (3.1)	7 (2.4)	6 (4.3)		10 (3.2)	2 (1.8)	1 (33.3)		10 (2.8)	3 (4.5)	0 (0.0)	
Unknown	118	75	43		86	31	1		101	17	0	
INSS stage, N(%)				0.3793 (Stage 4 vs. Non-Stage 4)				0.3480 (Stage 4 vs. Non-Stage 4)				0.4501* (Stage 4 vs. Non-Stage 4)
2A	1 (0.2)	1 (0.3)	0 (0.0)		1 (0.3)	0 (0.0)	0 (0.0)		1 (0.2)	0 (0.0)	0 (0.0)	
2B	6 (1.2)	3 (0.9)	3 (1.9)		4 (1.1)	2 (1.6)	0 (0.0)		4 (1.0)	2 (2.7)	0 (0.0)	
3	22 (4.5)	18 (5.5)	4 (2.5)		19 (5.4)	3 (2.3)	0 (0.0)		19 (4.6)	3 (4.1)	0 (0.0)	
4S	2 (0.4)	1 (0.3)	1 (0.6)		1 (0.3)	1 (0.8)	0 (0.0)		1 (0.2)	1 (1.4)	0 (0.0)	
4	454 (93.6)	302 (92.9)	152 (95.0)		329 (92.9)	122 (95.3)	3 (100.0)		385 (93.9)	68 (91.9)	1 (100.0)	
Unknown	59	36	23		45	13	1		49	10	0	

[a] * Indicates Fisher's Exact Test was used

© 2025 Umaretiya PJ et al. *JAMA Network Open.*

eFigure 1. Survival Curves for Induction/Consolidation, Post-Consolidation, and Relapse Cohorts by Household Poverty (Red = No household poverty; Blue = Household poverty). **A.** Induction/Consolidation five-year EFS: log-rank p-value= 0.14. **B.** Induction/Consolidation five-year OS: log-rank p-value= 0.037. **C.** Post-consolidation five-year EFS: log-rank p-value= 0.10. **D.** Post-consolidation five-year OS: log-rank p-value= 0.022. **E.** Relapse five-year OS: log-rank p-value= 0.0072.

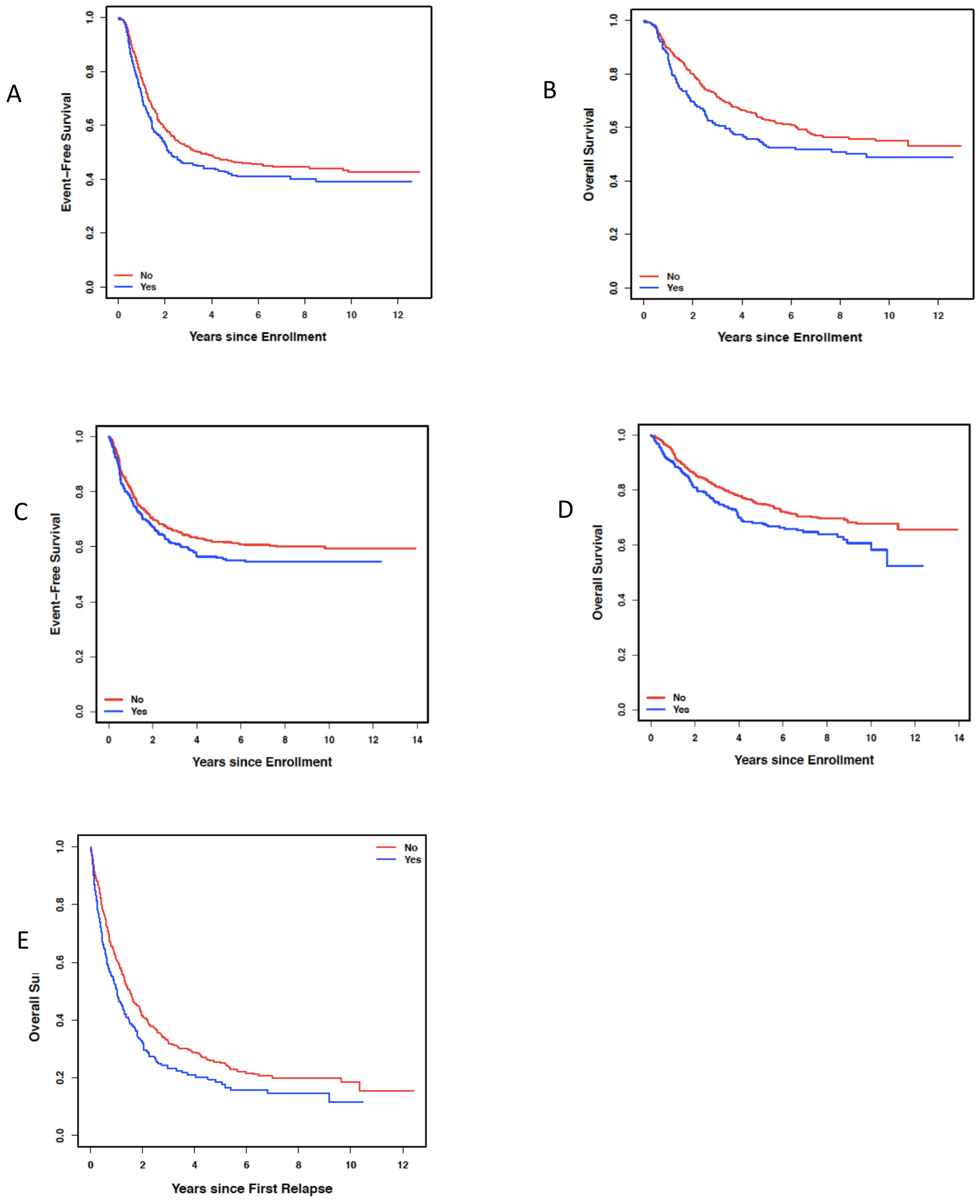

© 2025 Umaretiya PJ et al. *JAMA Network Open.*

eFigure 2. Survival Curves for Induction/Consolidation, Post-Consolidation, and Relapse Cohorts by Area Poverty (Red = No area poverty; Blue = Area poverty). **A.** Induction/Consolidation five-year EFS: log-rank p-value= 0.14. **B.** Induction/Consolidation log-rank p-value= 0.05. **C.** Post-Consolidation five-year EFS: log-rank p-value= 0.53. **D.** Post-Consolidation five-year OS: log-rank p-value= 0.15. **E.** Relapse five-year OS: log-rank p-value= 0.0694.

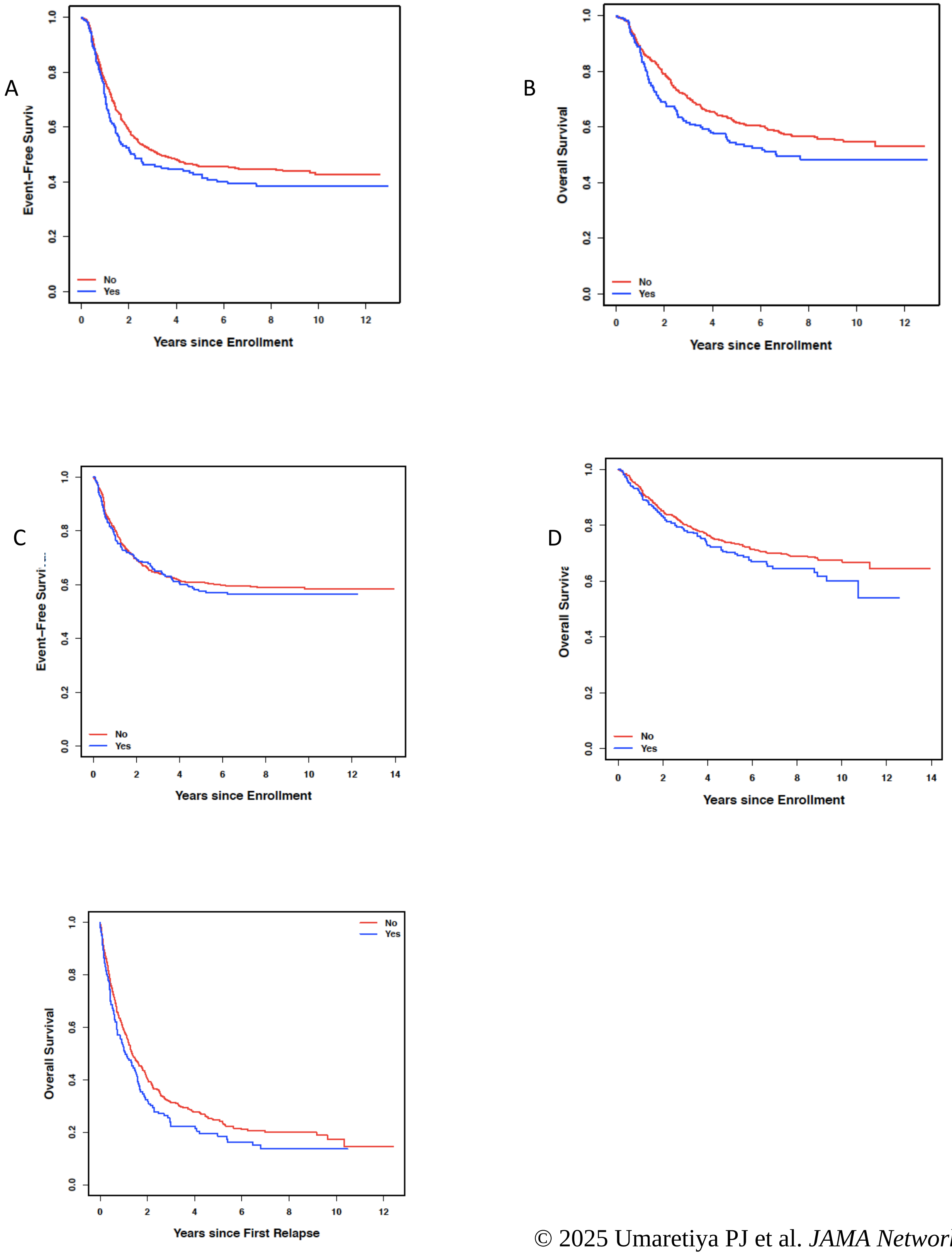

© 2025 Umaretiya PJ et al. *JAMA Network Open.*

eFigure 3. Survival Curves for Induction/Consolidation, Post-Consolidation, and Relapse Cohorts by Rurality. **A.** Induction/Consolidation five-year EFS: log-rank p-value= 0.76. **B.** Induction/Consolidation five-year OS: log-rank p-value= 0.50. **C.** Post-Consolidation five-year EFS: log-rank p-value= 0.80. **D.** Post-Consolidation five-year OS: log-rank p-value= 0.37. **E.** Relapse five-year OS: log-rank p-value= 0.0717.

A

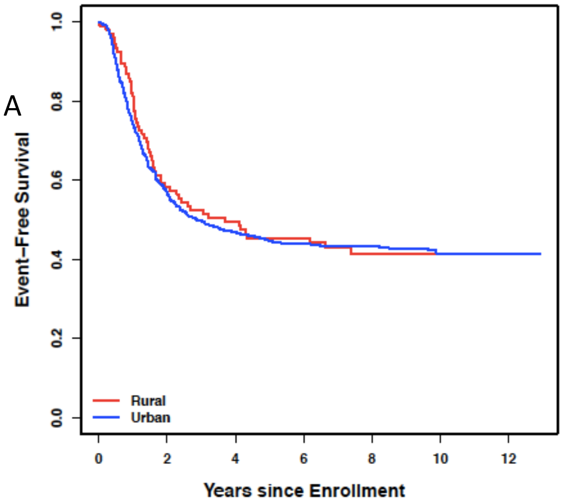

B

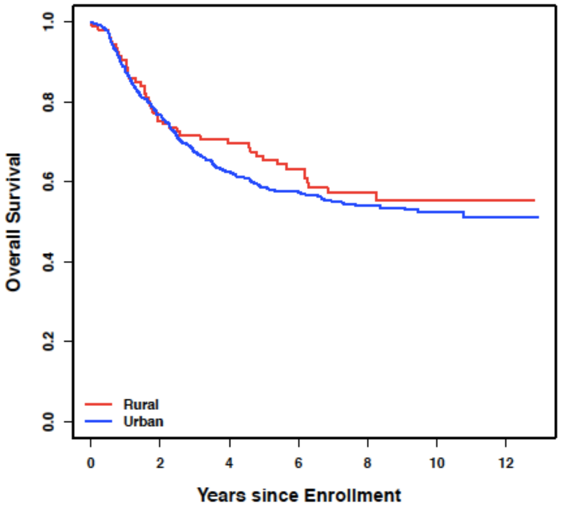

C

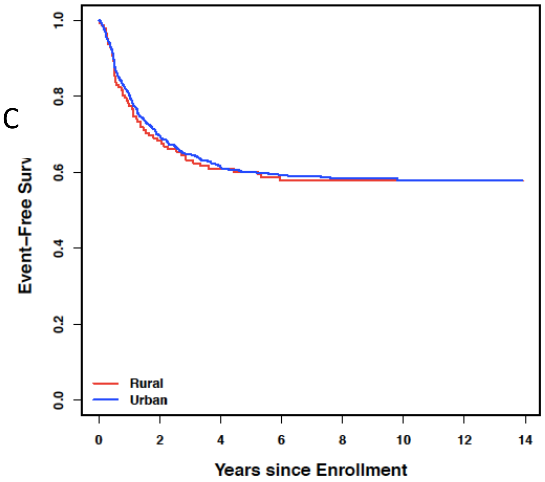

D

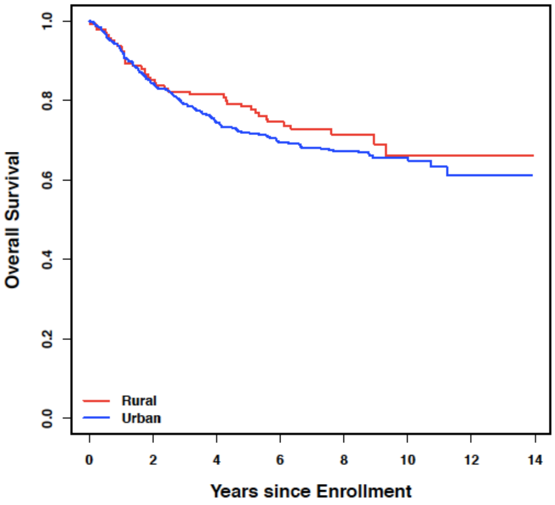

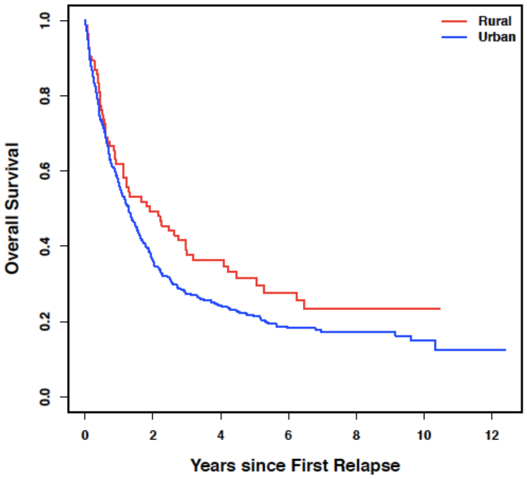

© 2025 Umaretiya PJ et al. *JAMA Network Open.*

**eTable 8.** Characteristics of Patients With Relapsed or Progressive Disease by Race and Ethnicity						
	Overall N=544	Non-Hispanic White N=366 (67.3)	Non-Hispanic Black N=88 (16.2)	Hispanic N=71 (13.1)	Non-Hispanic Other N=19 (3.5)	p-value[a]
Sociodemographic characteristics						
Age, N(%)						0.5093
< 18 mos	70 (12.9)	52 (14.2)	10 (11.4)	7 (9.9)	1 (5.3)	
≥18 mos	474 (87.1)	314 (85.8)	78 (88.6)	64 (90.1)	18 (94.7)	
Sex, N(%)						0.7199
Male	331 (60.8)	226 (61.7)	55 (62.5)	39 (54.9)	11 (57.9)	
Female	213 (39.2)	140 (38.3)	33 (37.5)	32 (45.1)	8 (42.1)	
Household poverty (insurance), N(%)						<0.0001
No	361 (66.4)	276 (75.4)	42 (47.7)	30 (42.3)	13 (68.4)	
Yes (public insurance)	183 (33.6)	90 (24.6)	46 (52.3)	41 (57.7)	6 (31.6)	
Area poverty, N(%)[b]						<0.0001
No	399 (73.9)	294 (81.0)	46 (52.9)	46 (64.8)	13 (68.4)	
Yes (≥ 20% of ZIP Code population living below FPL)	141 (26.1)	69 (19.0)	41 (47.1)	25 (35.2)	6 (31.6)	
Geographic location, N(%)[b]						0.0447
Rural	84 (15.5)	66 (18.1)	9 (10.2)	5 (7.0)	4 (21.1)	
Urban	459 (84.5)	299 (81.9)	79 (89.8)	66 (93.0)	15 (78.9)	
Disease characteristics						
Tumor *MYCN* status, N (%)[b]						0.1379
Amplified	189 (43.2)	133 (44.5)	23 (31.5)	27 (50.0)	6 (50.0)	
Not amplified	249 (56.8)	166 (55.5)	50 (68.5)	27 (50.0)	6 (50.0)	
Tumor histology, N(%)[b]						0.7249*
Unfavorable	413 (96.9)	281 (97.2)	66 (95.7)	55 (96.5)	11 (100.0)	
Favorable	13 (3.1)	8 (2.8)	3 (4.3)	2 (3.5)	0 (0.0)	
INSS stage, N(%)[b]						0.5826* [c]
2A	1 (0.2)	1 (0.3)	0 (0.0)	0 (0.0)	0 (0.0)	
2B	6 (1.2)	3 (0.9)	2 (2.4)	1 (1.6)	0 (0.0)	
3	22 (4.5)	15 (4.6)	2 (2.4)	5 (8.2)	0 (0.0)	
4	454 (93.6)	307 (93.6)	78 (95.1)	55 (90.2)	14 (100.0)	
4S	2 (0.4)	2 (0.6)	0 (0.0)	0 (0.0)	0 (0.0)	

[a]An * indicates that a Fisher's exact test was used
[b]4 with unknown area-level poverty; 1 with unknown geographic location; 106 with unknown *MYCN* status; 118 with unknown tumor histology; 59 with unknown INSS stage
[c]Non-stage 4 vs Stage 4

© 2025 Umaretiya PJ et al. *JAMA Network Open.*
