# Supplementary material for: Racial and Ethnic Survival Disparities Among Children With High-Risk Neuroblastoma: A Children’s Oncology Group Report
Source: JAMA Netw Open. 2025 Feb 14;8(2):e2458531. doi: 10.1001/jamanetworkopen.2024.58531 (PMC11829236; doi:10.1001/jamanetworkopen.2024.58531)
Supplement: Supplement 2. — Data Sharing Statement [file jamanetwopen-e2458531-s002.pdf]

Data Sharing Statement

Umaretiya. Racial and Ethnic Survival Disparities Among Children With High-Risk Neuroblastoma. *JAMA Netw Open*. Published February 07, 2025. doi:10.1001/jamanetworkopen.2024.58531

Data
Data available: No

Additional Information
Explanation for why data not available: Data to be made available upon reasonable request.
